# Discovery of (3-Benzyl-5-hydroxyphenyl)carbamates as New Antitubercular Agents with Potent In Vitro and In Vivo Efficacy

**DOI:** 10.3390/molecules24102021

**Published:** 2019-05-27

**Authors:** Ya-Juan Cheng, Zhi-Yong Liu, Hua-Ju Liang, Cui-Ting Fang, Niu-Niu Zhang, Tian-Yu Zhang, Ming Yan

**Affiliations:** 1 School of Pharmaceutical Sciences, Sun Yat-sen University, Guangzhou 510006, China; chengyj397563@163.com (Y.-J.C.); lianghj7@yeah.net (H.-J.L.); zhangniuniuu@yeah.net (N.-N.Z.); 2 State Key Laboratory of Respiratory Disease, Guangzhou Institutes of Biomedicine and Health, Chinese Academy of Science, Guangzhou 510530, China; liu_zhiyong@gibh.ac.cn (Z.-Y.L.); fang_cuiting@gibh.ac.cn (C.-T.F.); 3 University of Chinese Academy of Sciences (UCAS), Beijing 100049, China

**Keywords:** (3-benzyl-5-hydroxyphenyl)carbamate, *Mycobacterium tuberculosis*, antitubercular activity

## Abstract

A series of 3-amino-5-benzylphenol derivatives were designed and synthesized. Among them, (3-benzyl-5-hydroxyphenyl)carbamates were found to exert good inhibitory activity against *M. tuberculosis* H37Ra, H37Rv and clinically isolated multidrug-resistant *M. tuberculosis* strains (MIC = 0.625–6.25 μg/mL). The privileged compounds **3i** and **3l** showed moderate cytotoxicity against cell line A549. Compound **3l** also exhibited potent in vivo inhibitory activity on a mouse infection model via the oral administration. The results demonstrated 3-hydroxyphenylcarbamates as a class of new antitubercular agents with good potential.

## 1. Introduction

Tuberculosis (TB) is a dangerous disease caused by *M. tuberculosis* (Mtb). Today it still poses the serious threat to the human health. According to the report of World Health Organization (WHO), in 2018 there were more than 10.0 million people newly infected with Mtb worldwide [1] and the number of deaths among TB patients was greater than that of HIV patients. Efficient antitubercular agents were developed to fight TB. Typically, the treatment includes a combination of 2–4 antitubercular agents for 6–24 months. The long therapeutic period led to a poor compliance, which readily induced the drug resistance of Mtb. Multidrug-resistant tuberculosis (MDR-TB) and extensively drug-resistant TB (XDR-TB) were reported to spread over the world and brought new challenges for TB treatment [2,3,4]. In 2018, 560,000 people were infected with rifampin-resistant or MDR-TB. Among them, 230,000 patients died from the disease. In addition, over 30% patients infected with XDR-TB were not curable [5]. In the past 30 years, only bedaquiline [6] and delamanid [7] were marketed for the treatment of MDR-TB. Several candidates including pretomanid [8] and SQ109 [9], are still in the clinical study. Bedaquiline is toxic, mainly for causing irregular heart rhythm [10]. Furthermore, Mtb strains resistant to these new drugs have also been found [11,12]. The situation poses an urgent need for antitubercular agents with new structures and new mechanisms of action [13].

**Scheme 1:**
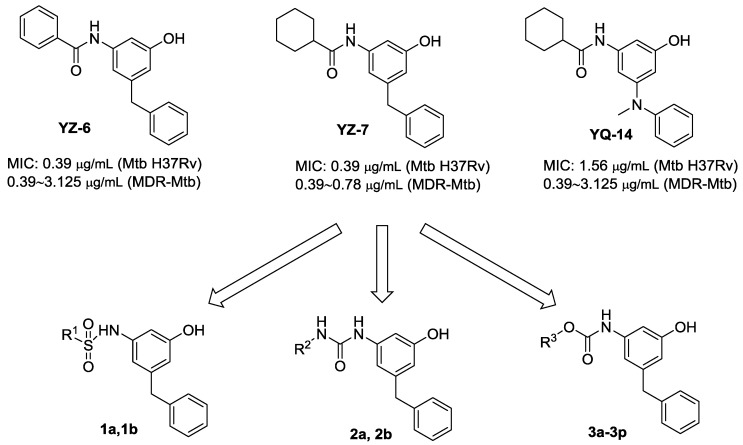
Design of new 3-amino-5-benzylphenol derivatives.

In the previous study, we found that *m*-amidophenol derivatives YZ-6 and YZ-7 showed potent in vitro inhibitory activity against Mtb H37Ra, H37Rv and clinically isolated MDR-Mtb strains (MIC = 0.39–3.125 μg/mL) (Scheme 1) [14,15]. However, the two compounds did not exert in vivo efficacy on a mouse infection model. Recently, we designed and synthesized a series of new *m*-amidophenol derivatives with 5-heteroatomic substitutents (OR, SR, NRR′) [16]. One privileged compound YQ-14 showed potent in vitro antitubercular activity and weak in vivo efficacy (Scheme 1). Because the clearance rates of YZ-6 and YQ-14 in mouse liver microsome were high (*T*_1/2_ = 6.60 and 5.43 min, respectively), further structural modifications to improve the metabolic stability and in vivo potency are desirable. We designed and synthesized new derivatives of YZ-6 and YZ-7 via the replacement of 1-amido group with sulfamide, urea and carbamate moieties. Such replacements were expected to provide a higher metabolic stability towards enzymatic hydrolysis. In addition, binding with the target may be enhanced considering the introduction of more oxygen and nitrogen atoms. Among the new derivatives, 3-hydroxylphenylcarbamates were found to exhibit potent in vitro and in vivo antitubercular efficacy. The results are reported in this paper.

## 2. Results and Discussion

### 2.1. Chemistry

**Scheme 2:**
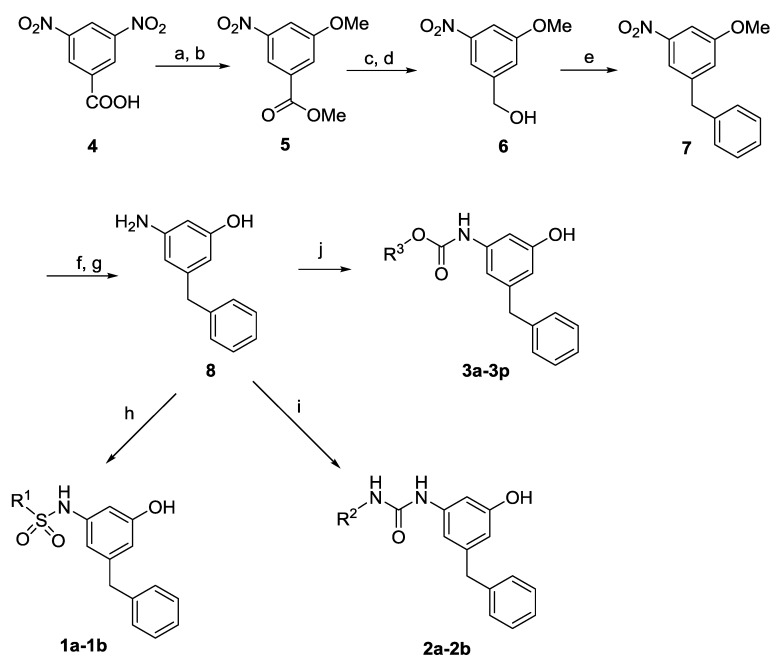
Synthesis of products **1a**–**1b**, **2a**–**2b** and **3a**–**3p**. Reagents and conditions: (a) SOCl_2_, CH_3_OH, reflux, 4 h, 60%; (b) MeOLi, MeOH, reflux, 12 h, 35%; (c) NaOH, CH_2_Cl_2_, CH_3_OH, rt, 3 h, 98%; (d) NaBH_4_, BF_3_.OEt_2,_ THF, 0 °C-rt, 1 h, 99%; (e) benzene, CH_2_Cl_2_, AlCl_3_, reflux, 5 h, 53%; (f) BBr_3_, CH_2_Cl_2_, −40 °C–0 °C, 12 h, 60%; (g) H_2_, 10% Pd/C, CH_2_Cl_2_/CH_3_OH, 4 h, 99%; (h) R^1^SO_2_Cl, Et_3_N, CH_2_Cl_2_, 0 °C-rt, 50–75%; (i) R^2^NCO, Et_3_N, CH_2_Cl_2_, rt, 50–75%; (j) R^3^OH, triphosgene, Et_3_N, CH_2_Cl_2_, 0 °C-rt, 20–50%.

The syntheses of 3-amino-5-benzylphenol derived sulfonamides **1a**–**1b**, ureas **2a**–**2b** and carbamates **3a**–**3p** are outlined in Scheme 2. 3-Amino-5-benzylphenol **8** was prepared via the reported procedure and used as the key intermediate [14]. Briefly, 3,5-dinitrobenzoic acid **4** was transformed to the methyl ester via the reflux in methanol with small amount of thionyl chloride. The subsequent substitution of nitro group with lithium methoxide afforded methyl 3-methoxy-5-nitro-benzoate **5**. The hydrolysis in aqueous sodium hydroxide and the reduction with NaBH_4_/BF_3_·OEt_2_ provided (3-methoxy-5-nitrophenyl)methanol **6**. Friedel-Crafts alkylation of compound **6** with benzene gave 1-benzyl-3-methoxy-5-nitrobenzene **7**. The subsequent demethylation and catalytic hydrogenation afforded compound **8**. The reaction of **8** with sulfonyl chlorides provided the products **1a**–**1b**. The treatment of **8** with isocyanates afforded the products **2a**–**2b**. The reaction of **8** with chloroformates provided the products **3a**–**3p** in good yields.

### 2.2. Antitubercular Activity against Mtb H37Ra

Antitubercular activity of **1a**–**1b**, **2a**–**2b** and **3a**–**3p** was assessed against avirulent autoluminescent Mtb H37Ra and the results are listed in Table 1 and Table 2. The growth of the bacteria was monitored by the bioluminescence intensity [17]. The minimal inhibitory concentrations (MIC) were determined as the lowest concentration at which the RLU was 90% lower than the drug-free control. Isoniazid (INH) was used as the positive control.

Benzenesulfonamide **1a** and cyclohexanesulfonamide **1b** almost did not show inhibitory activity against H37Ra (MIC = 50 μg/mL). However, phenylurea **2a** and cyclohexylurea **2b** showed higher inhibitory activity (MIC = 12.5 μg/mL and 6.25 μg/mL respectively).

The phenyl carbamate **3a** is much less active than its benzamide analog YZ-6 (MIC = 50 μg/mL vs. 5 μg/mL). The substitution on the phenyl ring (**3b**, **3c**) resulted in the loss of the inhibitory activity (MIC = 100 μg/mL). The benzyl carbamate **3d** showed better activity (MIC = 10 μg/mL). Furthermore, alkyl carbamates **3e**–**3m** were examined. The methyl carbamate **3e** had a low inhibitory activity (MIC = 25 μg/mL), but ethyl and *t*-butyl carbamates **3f**–**3g** showed improved activity (MIC = 5 μg/mL). The cycloalkyl carbamates (**3h**–**3j**) were found to be more potent. The cyclo-hexyl and cyclo-heptyl carbamates **3i** and **3j** exhibited good inhibitory activity (MIC = 1.25 μg/mL); 4-Methyl-cyclo-hexyl carbamate **3k** displayed a similar activity (MIC = 1.25 μg/mL), but 4,4-dimethyl-cyclo-hexyl carbamate **3l** afforded better inhibitory activity (MIC = 0.625 μg/mL). The substitution at cyclo-hexyl with bulkier 4-*tert*-butyl group (**3m**) decreased the inhibitory activity (MIC = 25 μg/mL). The substitution with hydrophilic group such as hydroxyl (**3n**) also decreased the inhibitory activity (MIC = 25 μg/mL). *N*-Methyl-piperidinyl and 4-pyridinylmethyl carbamates **3o** and **3p** lost the activity (MIC = 100 μg/mL). The results implicated that a hydrophobic moiety is important for the antitubercular activity. However, no general correlation between the MICs and CLogP values was observed.

In order to identify the key pharmacophores, we kept 4,4-dimethyl-*cyclo*-hexyl carbamate scaffold and replaced 1-hydroxyl with other functional groups. The inhibitory activity of compounds **3q** and **3r** against Mtb H37Ra are examined and the results are summarized in Table 3. The 1-carboxylic analog **3q** is inactive (MIC = 100 μg/mL), however the methoxy analog **3r** showed good inhibitory activity (MIC = 2.5 μg/mL).

### 2.3. In Vitro Inhibitory Activity against Mtb H37Rv and MDR-Mtb Strains

The compounds **3i** and **3l** were selected for the further evaluation of inhibitory activity against Mtb H_37_Rv and clinically isolated multidrug-resistant Mtb strains (MDR-Mtb). The results are summarized in Table 4. Good inhibitory activities (MIC = 2–8 µg/mL) were observed against Mtb H_37_Rv and MDR-Mtb strains K12, K16, K18, V4.

### 2.4. Evaluation of Cytotoxicity and Metabolic Stability

The cytotoxicity of **3i** and **3l** against human lung cancer cell line A549 was evaluated via MTT method and the results are summarized in Table 5. Moderate cytotoxicities were observed for compounds **3i** and **3l** (IC_50_ = 15.8 and 22.6 µg/mL). The selection indices (SI = IC_50_/MIC) are 12.6 and 36.2 respectively for **3i** and **3l**. The result demonstrated compound **3l** as a more suitable candidate for further evaluation.

The metabolic stability of **3l** in mouse liver microsome was examined. A quick clearance was observed (T_1/2_ = 1.76 min, CL = 1440 mL/min/gprot). The result indicated that the replacement of 1-amido group in the lead compounds YZ-6 and YZ-7 with carbamate moiety cannot improve the metabolic stability. The reason remains to be investigated.

### 2.5. In Vivo Inhibitory Activity against Mtb H37Ra

**Figure 1:**
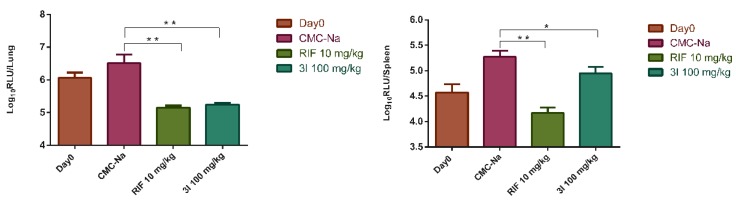
In vivo inhibitory activity of **3l** on a mouse model infected with Mtb H37Ra. Four mice per group were treated for 6 days. Data were presented as mean ± SD. ^*^, *p* < 0.05; ^**^, *p* < 0.001.

The in vivo efficacy of compound **3l** was evaluated in a mouse model infected with autoluminescent Mtb H37Ra. The compound **3l** was administered orally (100 mg/kg/day). The untreated control group was administered with sodium carboxymethyl cellulose (CMC-Na) solution. Rifampicin was used as the positive control. After five days treatment, the burdens of the bacteria in lungs and spleens of the mice were determined by the bioluminescence intensity detection. The results are summarized in Figure 1. A significant reduction (1.3 log) of relative light unit (RLU) in the lung was achieved compared with the untreated control group. A small reduction of RLU (0.4 log) was observed in the spleen.

## 3. Experimental

### 3.1. Chemistry

The NMR spectra of ^1^H and ^13^C were recorded on Bruker AVANCE 400 or 500 spectrometer (Karlsruhe, Germany). Chemical shifts of protons are reported in parts per million downfield from tetramethylsilane. Peaks are labeled as singlet (s), broad singlet (br), doublet (d), triplet (t), double doublet (dd), doublet of triplets (dt), multiplet (m). The high-resolution mass spectra were analyzed on a SHIMADZU LCMS-IT-TOF mass spectrometer (Tokyo, Japan). Melting points were determined in open capillary tubes on a MPA100 Optimelt automated melting point system. All chemicals were purchased from Sigma-Aldrich and Alfa Aesar chemical companies and were used without further purification. Compounds **4**–**8** were prepared according to the reported procedures [14].

#### 3.1.1. Synthesis of *N*-(3-benzyl-5-hydroxyphenyl)benzenesulfonamide (**1a**)

To a solution of 3-amino-5-benzylphenol **8** (119 mg, 0.6 mmol), DBU (9 mg, 0.06 mmol) in pyridine (2 mL) was added dropwise benzenesulfonyl chloride (211 mg, 1.2 mmol) at 0 °C. The reaction mixture was stirred at room temperature for 5 h. After water (30 mL) was added, the reaction mixture was extracted with EtOAc (10 mL × 3). The combined organic layer was dried over anhydrous Na_2_SO_4_, filtered and concentrated in vacuo. The residue was purified by column chromatography (petroleum ether/EtOAc = 4:1) to afford 1a as an orange solid (117 mg, 54% yield). M.p. 142–143 °C. ^1^H-NMR (400 MHz, DMSO) δ 10.10 (s, 1H), 9.37 (s, 1H), 7.75–7.66 (m, 2H), 7.64–7.58 (m, 1H), 7.52 (t, *J* = 7.5 Hz, 2H), 7.26 (t, *J* = 7.3 Hz, 2H), 7.21–7.15 (m, 1H), 7.12–7.00 (m, 2H), 6.41 (s, 1H), 6.38 (s, 1H), 6.24 (s, 1H), 3.71 (s, 2H). ^13^C-NMR (101 MHz, DMSO) δ 158.26, 143.53, 141.25, 140.02, 139.13, 133.23, 129.59, 129.07, 128.80, 127.14, 126.42, 112.05, 111.38, 105.19, 41.50. HRMS (ESI) calculated for C19H17NNaO3S+ [M + Na]^+^: 362.0788, found: 362.0821. (see Supplementary Materials)

#### 3.1.2. *N*-(3-Benzyl-5-hydroxyphenyl)cyclohexanesulfonamide (**1b**)

The compound was synthesized via a similar procedure of **1a**, using cyclohexanesulfonyl chloride (236.4 mg, 1.2 mmol) to replace benzenesulfonyl chloride. Orange solid (86 mg, 44% yield). M.p. 140–141 °C. ^1^H-NMR (400 MHz, DMSO) 1H-NMR (400 MHz, DMSO) δ 9.53 (s, 1H), 9.38 (s, 1H), 7.28 (t, *J* = 7.2 Hz, 2H), 7.23–7.12 (m, 3H), 6.54 (s, 1H), 6.52 (s, 1H), 6.30 (s, 1H), 3.79 (s, 2H), 2.97–2.84 (m, 1H), 2.05–1.89 (m, 2H), 1.80–1.67 (m, 2H), 1.64–1.52 (m, 1H), 1.45–1.31 (m, 2H), 1.22–1.05 (m, 3H). ^13^C-NMR (101 MHz, DMSO) δ 158.42, 143.71, 141.43, 140.06, 129.13,128.82, 126.44, 111.62, 110.91, 104.55, 59.30, 41.58, 26.42, 25.22, 24.89. HRMS (ESI) calculated for C_19_H_23_NO_3_S^+^ [M + H]^+^: 346.1471, found: 346.1473.

#### 3.1.3. Synthesis of 1-(3-benzyl-5-hydroxyphenyl)-3-phenylurea (**2a**)

To a solution of 3-amino-5-benzylphenol **8** (119 mg, 0.6 mmol), triethylamine (72 mg, 0.72 mmol) in THF (10 mL) was added dropwise phenylisocyanate (86 mg, 0.72 mmol) at 0 °C. The reaction mixture was stirred at room temperature overnight. After the reaction was quenched with water (30 mL), the mixture was extracted with EtOAc (10 mL × 3). The combined organic layer was dried over anhydrous Na_2_SO_4_, filtered and concentrated in vacuo. The residue was purified by column chromatography (petroleum ether/EtOAc = 4:1) to afford **2a** as a white solid (103 mg, 54% yield). M.p. 178–179 °C. ^1^H-NMR (400 MHz, DMSO) δ 9.27 (s, 1H), 8.53 (s, 1H), 8.51 (s, 1H), 7.47–7.38 (m, 2H), 7.33–7.16 (m, 7H), 6.95 (t, *J* = 7.3 Hz, 1H), 6.90 (t, *J* = 2.0 Hz, 1H), 6.64 (s, 1H), 6.24 (s, 1H), 3.81 (s, 2H). ^13^C-NMR (101 MHz, DMSO) δ 158.22, 152.81, 143.23, 141.62, 141.21, 140.18, 129.25, 128.84, 126.41, 122.22, 118.55, 109.99, 109.76, 103.46, 41.74. HRMS (ESI) calculated for C_20_H_18_N_2_O_2_^+^ [M + H]^+^: 319.1441, found: 319.1441.

#### 3.1.4. 1-(3-Benzyl-5-hydroxyphenyl)-3-cyclohexylurea (**2b**)

The compound was synthesized via a similar procedure of **2a**, using cyclohexanylisocyanate (90.1 mg, 0.72 mmol) to replace phenylisocyanate. White solid, (74 mg, 38% yield). M.p. 193–194 °C. ^1^H-NMR (400 MHz, DMSO) δ 9.13 (s, 1H), 8.12 (s, 1H), 7.34–7.24 (m, 2H), 7.22–7.13 (m, 3H), 6.79 (s, 1H), 6.53 (s, 1H), 6.14 (s, 1H), 5.94 (d, *J* = 7.8 Hz, 1H), 3.75 (s, 2H), 3.49–3.37 (m, 1H), 1.86–1.71 (m, 2H), 1.68–1.57 (m, 2H), 1.37–1.21 (m, 3H), 1.19–1.06 (m, 3H). ^13^C-NMR (400 MHz, DMSO) δ 158.11, 154.69, 142.95, 141.96, 141.69, 129.19, 128.78, 126.35, 109.21, 109.13, 102.85, 47.89, 41.78, 33.42, 25.71, 24.77. HRMS (ESI) calculated for C20H25N2O2^+^ [M + H]^+^: 325.1911, found:325.1891.

#### 3.1.5. Synthesis of Phenyl (3-benzyl-5-hydroxyphenyl)carbamate (**3a**)

To a solution of phenol (112 mg, 1.2 mmol), triphosgene (178 mg, 0.6 mmol) in CH_2_Cl_2_ (10 mL) was added dropwise triethylamine (121 mg, 1.2 mmol) at 0 °C. The reaction mixture was stirred for 0.5 h and then 3-amino-5-benzylphenol **8** (119 mg, 0.6 mmol) was added. The mixture was stirred at room temperature for 5 h. After the reaction was quenched with water (30 mL), the mixture was extracted with EtOAc (10 mL × 3). The combined organic layer was dried over anhydrous Na_2_SO_4_, filtered and concentrated in vacuo. The residue was purified by column chromatography (petroleum ether/EtOAc = 4:1) to afford **3a** as a white solid (61 mg, 32% yield). M.p. 175–176 °C. ^1^H-NMR (500 MHz, DMSO) δ 10.06 (s, 1H), 9.38 (s, 1H), 7.42 (t, *J* = 7.9 Hz, 2H), 7.32–7.25 (m, 3H), 7.22–7.17 (m, 5H), 6.87 (s, 1H), 6.84 (s, 1H) 6.31 (s, 1H), 3.80 (s, 2H). ^13^C-NMR (126 MHz, DMSO) δ 158.20, 152.01, 150.99, 143.30, 141.51, 140.04, 129.87, 129.17, 128.85, 126.43, 125.85, 122.44, 111.15, 110.27, 103.94, 41.77. HRMS (ESI) calculated for C_20_H_18_NO_3_^+^ [M + H]^+^: 320.1261, found: 320.1281.

#### 3.1.6. 4-Chlorophenyl (3-benzyl-5-hydroxyphenyl)carbamate (**3b**)

The compound was synthesized via a similar procedure of **3a**, using 4-chlorine phenol (154.3 mg, 1.2 mmol) to replace phenol. White solid (74 mg, 35% yield). M.p. 144–145 °C. ^1^H-NMR (400 MHz, DMSO) δ 10.10 (s, 1H), 9.37 (s, 1H), 7.50–7.43 (m, 2H), 7.31–7.26 (m, 2H), 7.26–7.22 (m, 2H), 7.22–7.16 (m, 3H), 6.84 (s, 1H), 6.81 (s, 1H), 6.31 (s, 1H), 3.78 (s, 2H). ^13^C-NMR (101 MHz, DMSO) δ 158.25, 151.69, 149.83, 143.25, 141.49, 139.87, 129.94, 129.75, 129.17, 128.84, 126.43, 124.32, 111.19, 110.29, 104.17, 41.77. HRMS (ESI) calculated for C_20_H_17_ClNO_3_^+^ [M + H]^+^: 354.0854, found: 354.0891.

#### 3.1.7. 3,5-Dimethylphenyl (3-benzyl-5-hydroxyphenyl)carbamate (**3c**)

The compound was synthesized via a similar procedure of **3a**, using 3, 5-dimethylphenol (146.5 mg, 1.2 mmol) to replace phenol. White solid (42 mg, 20% yield). M.p. 136–138 °C. ^1^H-NMR (400 MHz, DMSO) δ 9.95 (s, 1H), 9.31 (s, 1H),7.28 (t, *J* = 7.4 Hz, 2H), 7.22–7.15 (m, 3H), 6.87 (s, 1H), 6.83 (d, 2H), 6.78 (s, 2H), 6.29 (s, 1H), 3.79 (s, 2H), 2.27 (s, 6H). ^13^C-NMR (101 MHz, DMSO) δ 158.20, 152.11, 150.89, 143.30, 141.52, 140.05, 139.23, 129.16, 128.83, 127.24, 126.42, 119.89, 111.12, 110.28, 103.99, 41.80, 21.28. HRMS (ESI) calculated for C22H22NO3^+^ [M + H]^+^: 348.1568, found: 348.1594.

#### 3.1.8. Benzyl (3-benzyl-5-hydroxyphenyl)carbamate (**3d**)

The compound was synthesized via a similar procedure of **3a**, using benzyl alcohol (129.7 mg, 1.2 mmol) to replace phenol. White solid (94 mg, 47% yield). M.p. 124–125 °C. ^1^H-NMR (400 MHz, DMSO) δ 9.57 (s, 1H), 9.26 (s, 1H), 7.45–7.32 (m, 5H), 7.27 (t, *J* = 7.4 Hz, 2H), 7.22–7.14 (m, 3H), 6.83 (s, 1H), 6.78 (s, 1H), 6.24 (s, 1H), 5.11 (s, 2H), 3.77 (s, 2H). ^13^C-NMR (101 MHz, DMSO) δ 158.14, 153.71, 143.11, 141.55, 140.50, 137.20, 129.15, 128.89, 128.82, 128.45, 126.39, 103.77, 66.02, 41.81. HRMS (ESI) calculated for C21H20NO3+ [M + H]^+^: 334.1424, found: 334.1438.

#### 3.1.9. Methyl (3-benzyl-5-hydroxyphenyl)carbamate (**3e**)

The compound was synthesized via a similar procedure of **3a**, using methanol (38.5 mg, 1.2 mmol) to replace phenol. White solid (80 mg, 52% yield). M.p. 120–121 °C. ^1^H-NMR (400 MHz, DMSO) δ 9.42 (s, 1H), 9.24 (s, 1H), 7.28 (t, *J* = 7.4 Hz, 2H), 7.18 (m, 3H), 6.84 (s, 1H), 6.76 (s, 1H), 6.25 (s, 1H), 3.79 (s, 2H), 3.63 (s, 3H). ^13^C-NMR (101 MHz, DMSO) δ 158.13, 154.31, 143.05, 141.56, 140.57, 129.15, 128.81, 126.39, 110.58, 110.08, 103.73, 51.92, 41.80. HRMS (ESI) calculated for C_15_H_16_NO_3_^+^ [M + H]^+^: 258.1108, found: 258.1125.

#### 3.1.10. Ethyl (3-benzyl-5-hydroxyphenyl)carbamate (**3f**)

The compound was synthesized via a similar procedure of **3a**, using ethanol (55.5 mg, 1.2 mmol) to replace phenol. White solid (80 mg, 49% yield). M.p. 167–168 °C. ^1^H-NMR (400 MHz, DMSO) δ 9.40 (s, 1H), 9.23 (s, 1H), 7.27 (t, *J* = 7.4 Hz, 2H), 7.23–7.11 (m, 3H), 6.81 (s, 1H), 6.77 (s, 1H), 6.22 (s, 1H), 4.08 (q, *J* = 7.0 Hz, 2H), 3.76 (s, 2H), 1.21 (t, *J* = 7.2 Hz, 3H). ^13^C-NMR (101 MHz, DMSO) δ 158.09, 153.89, 143.02, 141.57, 140.64, 129.14, 128.81, 126.38, 110.52, 110.12, 103.76, 60.43, 41.81, 14.99. HRMS (ESI) calculated for C_16_H_18_NO_3_^+^ [M + H]^+^: 272.1263, found: 272.1281.

#### 3.1.11. tert-Butyl (3-benzyl-5-hydroxyphenyl)carbamate (**3g**)

The compound was synthesized via a similar procedure of **3a**, using tert-butanol (88.9 mg, 1.2 mmol) to replace phenol. White solid (72 mg, 38% yield). M.p. 158–159 °C. ^1^H-NMR (400 MHz, DMSO) δ 9.19 (s, 1H), 9.15 (s, 1H), 7.27 (t, *J* = 7.4 Hz, 2H), 7.20–7.15 (m, 3H), 6.80 (s, 1H), 6.78 (s, 1H), 6.18 (s, 1H), 3.76 (s, 2H), 1.44 (s, 9H). ^13^C-NMR (101 MHz, DMSO) δ 158.01, 153.12, 142.89, 141.65, 140.94, 129.13, 128.80, 126.36, 110.24, 110.01, 103.62, 79.23, 41.83, 28.60. HRMS (ESI) calculated for C_18_H_21_NNaO_3_^+^ [M + Na]^+^: 322.1394, found: 322.1414.

#### 3.1.12. Cyclopentyl (3-benzyl-5-hydroxyphenyl)carbamate (**3h**)

The compound was synthesized via a similar procedure of **3a**, using cyclopentanol (103.5 mg, 1.2 mmol) to replace phenol. White solid (80 mg, 43% yield). M.p. 122–123 °C. ^1^H-NMR (400 MHz, DMSO) δ 9.31 (s, 1H), 9.20 (s, 1H), 7.28 (t, *J* = 7.4 Hz, 2H), 7.23–7.14 (m, 3H), 6.81 (s, 1H), 6.78 (s, 1H), 6.22 (s, 1H), 5.11–4.98 (m, 1H), 3.76 (s, 2H), 1.92–1.78 (m, 2H), 1.74–1.61 (m, 4H), 1.61–1.51 (m, 2H). ^13^C-NMR (101 MHz, DMSO) δ 158.07, 153.70, 142.96, 141.60, 140.73, 129.14, 128.80, 126.37, 110.43, 110.07, 103.70, 76.95, 41.83, 32.77, 23.72. HRMS (ESI) calculated for C_19_H_22_NO_3_^+^ [M + H]^+^: 312.1573, found: 312.1594.

#### 3.1.13. Cyclohexyl (3-benzyl-5-hydroxyphenyl)carbamate (**3i**)

The compound was synthesized via a similar procedure of **3a**, using cyclohexanol (120.1 mg, 1.2 mmol) to replace phenol. White solid (74 mg, 38% yield). M.p. 156–157 °C. ^1^H-NMR (400 MHz, DMSO) δ 9.33 (s, 1H), 9.22 (s, 1H), 7.28 (t, *J* = 7.4 Hz, 2H), 7.22–7.14 (m, 3H), 6.82 (s, 1H), 6.79 (s, 1H), 6.23 (s, 1H), 4.66–4.53 (m, 1H), 3.76 (s, 2H), 1.91–1.80 (m, 2H), 1.77–1.64 (m, 2H), 1.57–1.46 (m, 1H), 1.44–1.23 (m, 5H). ^13^C-NMR (101 MHz, CDCl3) δ 162.82, 158.19, 147.73, 146.34, 145.48, 133.89, 133.55, 131.12, 115.18, 114.83, 108.44, 77.30, 46.58, 36.84, 30.15, 28.64. HRMS (ESI) calculated for C_20_H_24_NO_3_^+^ [M + H]^+^: 326.1737, found: 326.1751.

#### 3.1.14. Cycloheptyl (3-benzyl-5-hydroxyphenyl)carbamate (**3j**)

The compound was synthesized via a similar procedure of **3a**, using cycloheptanol (137.1 mg, 1.2 mmol) to replace phenol. White solid (83 mg, 41% yield). M.p. 133–134 °C. ^1^H-NMR (400 MHz, DMSO) δ 9.31 (s, 1H),7.28 (t, *J* = 7.4 Hz, 2H), 7.22–7.13 (m, 3H), 6.81 (s, 1H), 6.79 (s, 1H), 6.23 (s, 1H), 4.90–4.65 (m, 1H), 3.76 (s, 2H), 1.94–1.83 (m, 2H), 1.72–1.58 (m, 4H), 1.53 (s, 4H), 1.48–1.37 (m, 2H). ^13^C-NMR (101 MHz, DMSO) δ 158.07, 153.42, 142.96, 141.60, 140.78, 129.14, 128.80, 126.37, 110.41, 110.08, 103.70, 74.95, 41.85, 34.11, 28.22, 22.83. HRMS (ESI) calculated for C_21_H_26_NO_3_^+^ [M + H]^+^: 340.1937, found: 340.1907.

#### 3.1.15. 4-Methyl-cyclohexyl (3-benzyl-5-hydroxyphenyl)carbamate (**3k**)

The compound was synthesized via a similar procedure of 3a, using 4-Methylcyclohexanol (137.1 mg, 1.2 mmol) to replace phenol. White solid (89 mg, 44% yield). M.p. 113–114 °C. ^1^H-NMR (400 MHz, DMSO) δ 9.34 (s, 1H), 9.28 (s, 1H), 7.28 (t, *J* = 7.4 Hz, 2H), 7.23–7.15 (m, 3H), 6.95–6.72 (m, 2H), 6.24 (s, 1H), 4.81 (s, 1H), 4.49 (s, 1H), 3.77 (d, *J* = 3.0 Hz, 2H), 1.99–1.88 (m, 1H), 1.82–1.67 (m, 2H), 1.60–1.42 (m, 3H), 1.35–1.26 (m, 2H), 1.09–0.96 (m, 1H), 0.94–0.85 (m, 3H). ^13^C-NMR (101 MHz, DMSO) δ 158.07, 153.55, 153.44, 142.99, 141.59, 140.77, 140.70, 129.13, 128.80, 126.38, 110.41, 110.13, 103.74, 73.37, 69.68, 41.85, 33.07, 32.10, 31.65, 31.08, 29.63, 29.57, 22.34, 22.18. HRMS (ESI) calculated for C_21_H_25_NNaO_3_^+^ [M + Na]^+^: 362.1713, found: 362.1727.

#### 3.1.16. 4,4-Dimethyl-cyclohexyl (3-benzyl-5-hydroxyphenyl)carbamate (**3l**)

The compound was synthesized via a similar procedure of **3a**, using 4,4-Dimethylcyclohexanol (153.8 mg, 1.2 mmol) to replace phenol. White solid (104 mg, 49% yield). M.p. 129–130 °C. ^1^H-NMR (400 MHz, DMSO) δ 9.34 (s, 1H), 9.23 (s, 1H), 7.28 (t, *J* = 7.4 Hz, 2H), 7.22–7.14 (m, 3H), 6.81 (s, 1H), 6.79 (s, 1H), 6.23 (s, 1H), 4.68–4.49 (m, 1H), 3.76 (s, 2H), 1.84–1.67 (m, 2H), 1.59–1.47 (m, 2H), 1.47–1.38 (m, 2H), 1.32–1.19 (m, 2H), 0.92 (s, 3H), 0.91 (s, 3H). ^13^C-NMR (101 MHz, DMSO) δ 157.98, 153.44, 142.91, 141.59, 140.74, 129.14, 128.79, 126.37, 110.45, 110.11, 103.86, 72.42, 41.85, 36.27, 29.75, 27.67. HRMS (ESI) calculated for C22H28NO3^+^ [M + H]^+^: 354.2050, found: 354.2064.

#### 3.1.17. 4-(tert-Butyl)-cyclohexyl (3-benzyl-5-hydroxyphenyl)carbamate (**3m**)

The compound was synthesized via a similar procedure of **3a**, using 4-tert-Butylcyclohexanol (187.5 mg, 1.2 mmol) to replace phenol. White solid (84 mg, 37% yield). M.p. 136–137 °C. ^1^H-NMR (600 MHz, DMSO) δ 9.38 (s, 1H), 9.24 (s, 1H), 7.28 (t, *J* = 7.5 Hz, 2H), 7.20–7.16 (m, 3H), 6.81 (s, 1H), 6.79 (s, 1H), 6.22 (s, 1H), 4.51–4.43 (m, 1H), 3.76 (s, 2H), 2.06–1.97 (m, 2H), 1.82–1.70 (m, 2H), 1.33–1.24 (m, 2H), 1.12–1.05 (m, 2H), 1.02–0.99 (m, 1H), 0.85 (s, 9H). ^13^C-NMR (101 MHz, DMSO) δ 158.07, 153.42, 142.95, 141.59, 140.72, 129.14, 128.79, 126.37, 110.44, 110.07, 103.70, 73.59, 46.99, 41.83, 32.60, 32.51, 27.94, 25.55. HRMS (ESI) calculated for C_24_H_32_NO_3_^+^ [M + H]^+^: 382.2365, found: 382.2377.

#### 3.1.18. 4-Hydroxycyclohexyl (3-benzyl-5-hydroxyphenyl)carbamate (**3n**)

The compound was synthesized via a similar procedure to **3a**, using 1,4-Cyclohexanediol (139.4 mg, 1.2 mmol) to replace phenol. Sticky liquid (67 mg, 33% yield). ^1^H-NMR (400 MHz, DMSO) δ 9.34 (s, 1H), 9.23 (s, 1H), 7.28 (t, *J* = 7.4 Hz, 2H), 7.23–7.14 (m, 3H), 6.88–6.76 (m, 2H), 6.24 (s, 1H), 4.73–4.45 (m, 2H), 3.77 (s, 2H), 3.69–3.45 (m, 1H), 2.03–1.89 (m, 1H), 1.88–1.71 (m, 2H), 1.58 (t, *J* = 8.6 Hz, 3H), 1.45–1.21 (m, 2H). ^13^C-NMR (101 MHz, CDCl3) δ 162.81, 158.17, 147.76, 146.35, 145.50, 145.43, 133.90, 133.56, 131.13, 115.14, 114.77, 108.37, 77.11, 75.65, 72.49, 70.43, 46.57, 37.51, 35.61, 34.21, 32.30. HRMS (ESI) calculated for C_20_H_23_NNaO_4_^+^[M + Na]^+^: 364.1519, found: 364.1512.

#### 3.1.19. 1-Methylpiperidin-4-yl (3-benzyl-5-hydroxyphenyl)carbamate (**3o**)

The compound was synthesized via a similar procedure of **3a**, using N-Methyl-4-piperidinol (138.2 mg, 1.2 mmol) to replace phenol. Sticky liquid (38 mg, 19% yield). ^1^H-NMR (400 MHz, DMSO) δ 9.41 (s, 1H), 9.23 (s, 1H), 7.28 (t, *J* = 7.4 Hz, 2H), 7.23–7.12 (m, 3H), 6.82 (s, 1H), 6.79 (s, 1H), 6.24 (s, 1H), 4.68–4.53 (m, 1H), 3.76 (s, 2H), 2.70–2.57 (m, 2H), 2.23–2.11 (m, 5H), 1.93–1.81 (m, 2H), 1.68–1.53 (m, 2H). ^13^C-NMR (101 MHz, DMSO) δ 158.09, 153.30, 143.01, 141.58, 140.64, 129.14, 128.81, 126.38, 110.51, 110.08, 103.72, 70.24, 53.05, 46.16, 41.83, 31.30. HRMS (ESI) calculated for C_20_H_24_N_2_O_3_^+^ [M + H]^+^: 341.1860, found: 341.1866.

#### 3.1.20. Pyridin-4-ylmethyl (3-benzyl-5-hydroxyphenyl)carbamate (**3p**)

The compound was synthesized via a similar procedure of **3a**, using 4-Pyridylcarbinol (130.9 mg, 1.2 mmol) to replace phenol. White solid (72 mg, 36% yield). M.p. 164–165 °C. ^1^H-NMR (400 MHz, DMSO) δ 9.70 (s, 1H), 9.28 (s, 1H), 8.57 (d, *J* = 5.6 Hz, 2H), 7.38 (d, *J* = 5.4 Hz, 2H), 7.28 (t, *J* = 7.4 Hz 2H), 7.25–7.15 (m, 3H), 6.85 (s, 1H), 6.78 (s, 1H), 6.27 (s, 1H), 5.17 (s, 2H), 3.81 (s, 2H). ^13^C-NMR (101 MHz, DMSO) δ 158.10, 153.40, 150.12, 146.43, 143.18, 141.53, 140.30, 129.17, 128.82, 126.41, 122.06, 110.85, 110.09, 103.75, 64.24, 41.78. HRMS (ESI) calculated for C_20_H_19_N_2_O_3_^+^ [M + H]^+^: 335.1367, found: 335.1390.

#### 3.1.21. 3-Benzyl-5-((((4,4-dimethylcyclohexyl)oxy)carbonyl)amino)benzoic acid (**3q**)

To a solution of 4,4-dimethylcyclohexan-1-ol (154 mg, 1.2 mmol), triphosgene (178 mg, 0.6 mmol) in CH_2_Cl_2_ (10 mL) was added dropwise triethylamine (121 mg, 1.2 mmol) at 0 °C. The reaction mixture was stirred for 0.5 h and then methyl 3-amino-5-benzylbenzoate (144 mg, 0.6 mmol) was added. The mixture was stirred at room temperature for 5 h. After the reaction was quenched with water (30 mL), the mixture was extracted with EtOAc (10 mL × 3). The combined organic layer was dried over anhydrous Na_2_SO_4_, filtered and concentrated in vacuo. The residue was purified by column chromatography (petroleumether/EtOAc = 4:1) to afford methyl 3-benzyl-5-((((4,4-dimethylcyclohexyl)oxy)carbonyl)amino)benzoate (59 mg, 25% yield).

To a solution of methyl 3-benzyl-5-((((4,4-dimethylcyclohexyl)oxy)carbonyl)amino)benzoate (59 mg, 0.15 mmol) in a mixed solvent (CH_2_Cl_2_/CH_3_OH = 9:1(*v*/*v*), 2 mL), was added sodium hydroxide (20 mg, 0.5 mmol). The reaction mixture was stirred at room temperature for 3 h. After the solvent was removed in vacuo, the residue was diluted with water (10 mL). The solution was extracted with diethyl ether (10 mL). The aqueous phase was cooled in an ice-bath, acidified to pH 2–3 with 1M HCl and extracted with AcOEt (10 mL × 2). The combined extracts were dried over anhydrous Na_2_SO_4_, filtered, and concentrated in vacuo to afford **3q** as a white solid (51 mg, 89% yield). M.p. 193–194 °C. ^1^H-NMR (500 MHz, DMSO) δ 12.89 (s, 1H), 9.71 (s, 1H), 7.95 (s, 1H), 7.60 (s, 1H), 7.43 (s, 1H), 7.33–7.27 (m, 2H), 7.24–7.18 (m, 3H), 4.62 (s, 1H), 3.95 (s, 2H), 1.76 (s, 2H), 1.59–1.50 (m, 2H), 1.46–1.39 (m, 2H), 1.29–1.22 (m, 2H), 0.91 (d, *J* = 7.4 Hz, 6H). ^13^C-NMR (126 MHz, DMSO) δ 167.77, 153.57, 142.60, 141.17, 140.12, 132.12, 129.21, 128.97, 128.77, 126.59, 123.95, 123.00, 117.38, 72.78, 41.43, 36.20, 29.77, 27.66. HRMS (ESI) calculated for C_23_H_28_NO_4_^+^ [M + H]^+^: 382.2013, found: 382.2011.

#### 3.1.22. 4,4-Dimethylcyclohexyl (3-benzyl-5-methoxyphenyl)carbamate (**3r**)

A mixture of **3l** (35 mg, 0.1 mmol), iodomethane (9.3 µL, 0.15 mmol) and anhydrous K_2_CO_3_ (27 mg, 0.2 mmol) in acetone (2 mL) was refluxed for 12 h. The mixture was cooled to room temperature and the solvent was removed in vacuo. The residue was purified by column chromatography over silica gel (hexane/EtOAc = 9:1) to give **3r** as a white solid (26 mg, 71% yield). M.p. 193–194 °C. ^1^H-NMR (400 MHz, DMSO) δ 9.46 (s, 1H), 7.28 (t, *J* = 7.4 Hz, 2H), 7.22–7.15 (m, 3H), 6.96 (s, 1H), 6.91 (s, 1H), 6.45 (s, 1H), 4.65–4.52 (m, 1H), 3.84 (s, 2H), 3.67 (s, 3H), 1.80–1.71 (m, 2H), 1.57–1.47 (m, 2H), 1.46–1.38 (m, 2H), 1.27–1.22 (m, 2H), 0.91 (d, *J* = 4.2 Hz, 6H). ^13^C-NMR (101 MHz, DMSO) δ 160.07, 153.45, 143.25, 141.47, 140.91, 129.10, 128.84, 126.44, 111.43, 108.90, 102.13, 72.54, 55.34, 41.86, 36.22, 29.78, 27.68. HRMS (ESI) calculated for C_23_H_30_NO_3_^+^ [M + H]^+^: 368.2222, found: 368.2220.

### 3.2. Biological Assays

#### 3.2.1. Determination of Minimum Inhibitory Concentration (MIC)

Minimum inhibitory concentration against Mtb H37Ra, H37Rv, and MDR-Mtb strains (provided by Guangzhou Chest Hospital) were determined via previously reported procedures [14].

#### 3.2.2. Evaluation of Cytotoxicity

The cytotoxicities of compounds **3i** and **3l** were assayed against human lung cancer cell line A549 at concentrations from 100 to 6.25 μg/mL. Approximately 4 × 10^4^ cells, suspended in culture medium, were seeded in the 96-well plate and then allowed to recover for 24 h at 37 °C under 5% CO_2_. The solution of tested compound was added to the plate and the final concentrations were 100 mg/mL, 50 mg/mL, 25 mg/mL, 12.5 mg/mL, and 6.25 mg/mL, respectively. Each experiment was repeated three times. After being incubated for 72 h, Fresh MTT was added to each well at a final concentration of 0.5 mg/mL and incubated with cells at 37 °C for 4 h. The formazan crystals were dissolved in 150 µL DMSO per each well. The absorbance at 490 nm was measured by microplate microscopy, and the survival rate was calculated by the formula (OD in the experimental group − blank group)/(OD in the cell group − blank group) × 100%. The cytotoxicity is shown as IC_50_ value, which was calculated by GraphPad Prism Software version 5 [18].

#### 3.2.3. Determination of the Clearance Rate in Mouse Liver Microsome

The mouse liver microsome was purchased from Xenotech company. The liver microsome was incubated in a 96-well plate. The final incubation volume was set at 45 μL, contained NADPH (2 mM), microsomes (0.5 mg/mL), the compound **3l** (1 μM), MgCl_2_ (5.0 mM), DMSO (0.01%) and BSA (0.005%). The plate was incubated at 37 °C for ten minutes, and then was added NADPH to trigger the reaction. The incubation times were 0, 5, 15, 30, 45 and 60 min. Ice-cold acetonitrile was added to terminate the reaction. The mixture was centrifuged at 4 °C for 15 min at 4000 rpm. The concentration of the compound **3l** in supernatant was determined by LC-MS/MS. The incubation time was plotted with the logarithm of the drug residual rate in the incubation system. k was calculated by linear regression, and the clearance rate was calculated based the formula ${\mathrm{Cl}}_{\mathrm{int}}=\frac{1000\times slope}{P}$ [19].

#### 3.2.4. In Vivo Inhibitory Activity against Mtb H37a

The experimental program involving animal feeding and use was submitted to, and approved by, the Animal Welfare Department of Guangzhou Institute of Biomedicine and Health, Chinese Academy of Sciences.

Mtb H37Ra isolated on plates were homogenized with sterile glass beads in a 250 mL flask containing 50 mL 7H9 with tween80. When RLU reached 2 million/mL, the broth culture was used to infect 4–6-week-old male BALB/c mice by tail vein injection. The day after infection (day 0), RLU counts were determined. The mice were first anesthetized by isoflurane inhalation and the RLU count was determined by laying the breast of the mouse on the detection hole of the luminometer and measuring light production twice for 3 s. Mice with similar RLU readings were randomly allocated to treatment groups (4 mice/group) and individually marked. The treatment groups received: 0.5% CMC-Na alone as negative control; RIF (10 mg/kg) as positive control; **3l** (100 mg/kg). The treatment was administered once daily by oral gavage for six days. On the final day of treatment, animals were sacrificed by cervical dislocation. The removed lungs and spleens were homogenized under sterile conditions in a 2 mL volume of PBS. Finally, RLU was detected for each homogenate.

## 4. Conclusions

In summary, we designed and synthesized a series of new 3-amino-5-benzylphenol derivatives. Several (3-benzyl-5-hydroxyphenyl)carbamates exerted potent in vitro inhibitory activity against Mtb H37Ra, H37Rv and clinically isolated multidrug-resistant Mtb strains. The introduction of hydrophobic carbocycles in the carbamate unit improved the antitubercular activity. The selected compounds **3i** and **3l** exhibited moderate cytotoxicities against tested cell line. The compound **3l** showed good oral efficacy on a mouse infection model. The significant reduction of the bacteria count in the lung was achieved. The further structural optimizations toward the increase of the metabolic stability and in vivo efficacy are currently under investigation.

## Figures and Tables

**Table 1:** Inhibitory activity of **1a**–**1b** and **2a**–**2b** against Mtb H37Ra.

Compound		MIC (µg/mL)	CLogP ^a^
**1a**	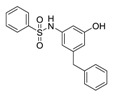	50	3.98
**1b**	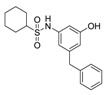	50	4.38
**2a**	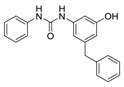	12.5	4.41
**2b**	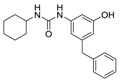	6.25	4.53
INH		0.1	/

**^a^** CLogP was calculated with ChemBioDraw Ultra 12.0.

**Table 2:**
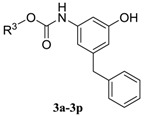
Inhibitory activity of **3a**–**3p** against Mtb H37Ra.

Compound	R^3^	MIC (µg/mL)	CLogP ^a^
**3a**	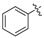	50	4.67
**3b**	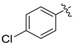	100	5.52
**3c**	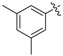	100	5.68
**3d**	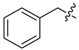	10	3.63
**3e**	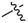	25	3.01
**3f**	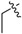	5	3.54
**3g**	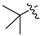	5	4.24
**3h**	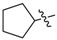	2.5	4.48
**3i**	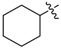	1.25	5.04
**3j**	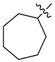	1.25	5.60
**3k** ^b^	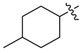	1.25	5.56
**3l**	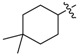	0.625	6.08
**3m** ^b^	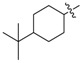	25	6.88
**3n** ^b^	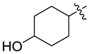	25	2.95
**3o**	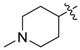	100	3.06
**3p**	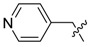	100	3.33
**INH**		0.1	

**^a^** CLogP was calculated with ChemBioDraw Ultra 12.0. ^b^ The compound was obtained as a mixture of *trans*- and *cis*-isomers.

**Table 3:** In vitro inhibitory activity of **3q**–**3r** against Mtb H37Ra.

Compound		MIC (μg/mL)	CLogP ^a^
**3q**	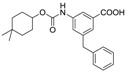	100	6.73
**3r**	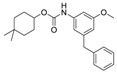	2.5	6.79
**INH**		0.1	

**^a^** CLogP was calculated with ChemBioDraw Ultra 12.0.

**Table 4:** Inhibitory activity (MIC, µg/mL) of compounds **3i** and **3l** against Mtb H_37_Rv and MDR-Mtb strains.

Mtb Strain	3i	3l	RIF	INH
H37Rv	4	4	0.03	0.41
K12	2	4	>10	>10
K16	2	4	>10	>10
K18	2	4	>10	>10
V4	4	8	>1	>10

RIF: rifampicin; INH: isoniazid.

**Table 5:** Cytotoxicity of compounds **3i** and **3l** against cell line A549.

Compounds	IC_50_ (µg/mL)	MIC (µg/mL, H37Ra)	SI
**3i**	15.8	1.25	12.6
**3l**	22.6	0.625	36.2

## Supplementary Materials

The spectra of ^1^H-NMR and ^13^C-NMR for the target compounds are available online.

## References

[1] World Health Organization (2018). WHO Global Tuberculosis Report 2018.

[2] Zumla A., Raviglione M., Hafner R., Fordham V.R.C. (2013). Tuberculosis. N. Engl. J. Med..

[3] Mishra R., Shukla P., Huang W., Hu N. (2015). Gene mutations in *Mycobacterium tuberculosis*: Multidrug-resistant TB as an emerging global public health crisis. Tuberculosis.

[4] Millard J., Ugarte-Gil C., Moore D.A.J. (2015). Multidrug resistant tuberculosis. Br. Med. J..

[5] Giffin R., Robinson S., Giffin R. (2009). Addressing the Threat of Drug-Resistant Tuberculosis: A Realistic Assessment of the Challenge. Workshop Summary of Institute of Medicine of the National Academies.

[6] Andries K., Verhasselt P., Guillemont J., Göhlmann W.H., Neefs J.M., Winkler H., Gestel J.V., Timmerman P., Zhu M., Lee E. (2005). A diarylquinoline drug active on the ATP synthase of *Mycobacterium tuberculosis*. Science.

[7] Matsumoto M., Hashizume H., Tomishige T., Kawasaki M., Tsubouchi H., Sasaki H., Shimokawa Y., Komatsu M. (2006). OPC-67683, a nitro-dihydroimidazooxazole derivative with promising action against tuberculosis in vitro and in mice. PLoS. Med..

[8] Stover C.K., Warrener P., VanDevanter D.R., Sherman D.R., Arain T.M., Langhorne M.H., Anderson S.W., Towell J.A., Yuan Y., McMurray D.N. (2000). A small-molecule nitroimidazopyran drug candidate for the treatment of tuberculosis. Nature.

[9] Sacksteder K.A., Protopopova M., Barry C.E., Andries K., Nacy C.A. (2012). Discovery and development of SQ109: A new antitubercular drug with a novel mechanism of action. Future Microbiol..

[10] Cox E., Laessig K. (2014). FDA approval of bedaquiline —the benefit—risk balance for drug-resistant tuberculosis. N. Engl. J. Med..

[11] Bloemberg G.V., Keller P.M., Stucki D., Trauner A., Borrell S., Latshang T., Coscolla M., Rothe T., Homke R., Ritter C. (2015). Acquired Resistance to Bedaquiline and Delamanid in Therapy for Tuberculosis. N. Engl. J. Med..

[12] Zhang S., Chen J.Z., Cui P., Shi W.L., Shi X.H., Niu H.X., Chan D., Yew W.W., Zhang W.H., Zhang Y. (2016). *Mycobacterium Tuberculosis* Mutations Associated with Reduced Susceptibility to Linezolid. Antimicrob. Agents Chemother..

[13] Dartois V., Barry C.E. (2013). A medicinal chemists’ guide to the unique difficulties of lead optimization for tuberculosis. Bioorg. Med. Chem. Lett..

[14] Zhang N.N., Liu Z.Y., Liang J., Tang Y.X., Qian L., Gao Y.M., Zhang T.Y., Yan M. (2018). Discovery of *m*-Amidophenol Derivatives as A New Class of Antitubercular Agents. Med. Chem. Commun..

[15] Yan M., Zhang T.Y., Zhang N.N., Liu Z.Y., Qian L., Tang Y.X., Cheng Y.J., Zhang X.J. (2018). *m*-Disubstituted phenol derivatives, their preparative methods and antitubercular applications. Patent CN.

[16] Zhang N.N., Tang Y.X., Qian L., Gao Y.M., Liu Z.Y., Zou Z.L., Zhang T.Y., Yan M. (2019). Design, synthesis and antitubercular activity of 3-amidophenols with 5-heteroatomic substitutions. Arch Pharm. Chem. Life Sci..

[17] Zhang T.Y., Li S.Y., Nuermberger E.L. (2012). Autoluminescent Mycobacterium tuberculosis for rapid, real-time, non-invasive assessment of drug and vaccine efficacy. PLoS ONE.

[18] Mustafa J., Khan S.I., Ma G., Walker L.A., Khan I.A. (2004). Synthesis and anticancer activities of fatty acid analogs of podophyllotoxin. Lipids.

[19] Bi H.C., Zuo Z., Chen X., Xu C.S., Wen Y.Y., Sun H.Y., Zhao L.Z., Pan Y., Deng Y., Liu P.Q. (2008). Preclinical factors affecting the pharmacokinetic behaviour of tanshinone IIA, an investigational new drug isolated from Salvia miltiorrhiza for the treatment of ischaemic heart diseases. Xenobiotica.

