# Supplementary material for: Discovery of (3-Benzyl-5-hydroxyphenyl)carbamates as New Antitubercular Agents with Potent In Vitro and In Vivo Efficacy
Source: Molecules. 2019 May 27;24(10):2021. doi: 10.3390/molecules24102021 (PMC6572244; doi:10.3390/molecules24102021)
Supplement: Supplementary file 1 [file molecules-24-02021-s001.pdf]

Discovery of 3-hydroxylphenylcarbamates as new antitubercular agents with potent *in vitro* and *in vivo* efficacy

Ya-juan Cheng[a]#, Zhi-yong Liu[b]#, Hua-ju Liang[a], Niu-niu Zhang[a], Tian-yu Zhang[b, c]*, Ming Yan[a]*

# These two authors contribute equally to this study.

[a] School of Pharmaceutical Sciences, Sun Yat-sen University, Guangzhou 510006, China.

[b] State Key Laboratory of Respiratory Disease, Guangzhou Institutes of Biomedicine and Health, Chinese Academy of Science, Guangzhou 510530, China.

[c] University of Chinese Academy of Sciences (UCAS), Beijing 100049, China

*Corresponding authors, e-mail: zhang_tianyu@gibh.ac.cn (T. Y. Zhang); yanming@mail.sysu.edu.cn (M. Yan)

Supporting Information

1. Copies of NMR spectra of products

Compound **1a**

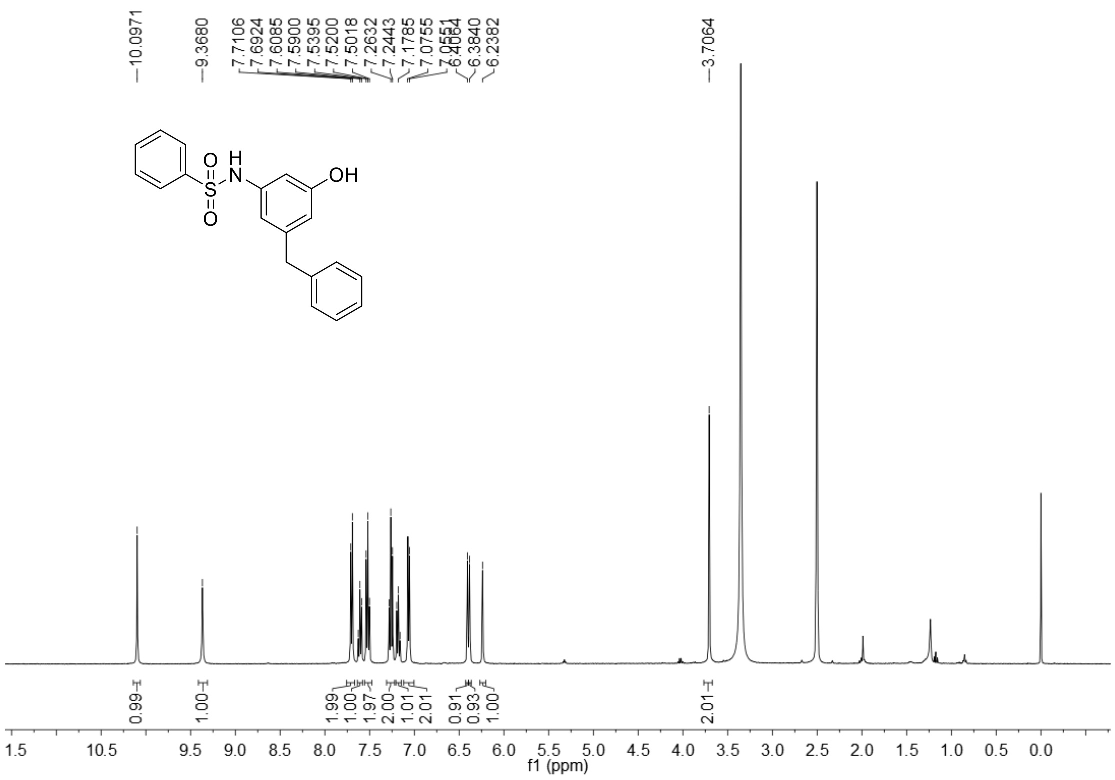
10.0971
9.3680
7.7106
7.6924
7.6085
7.5900
7.5395
7.5200
7.5018
7.2632
7.2443
7.1785
7.0755
7.0551
6.4064
6.3840
6.2382
3.7064
OH
0.99
1.00
1.99
1.00
1.97
2.00
1.01
2.01
0.91
0.93
1.00
2.01
f1 (ppm)

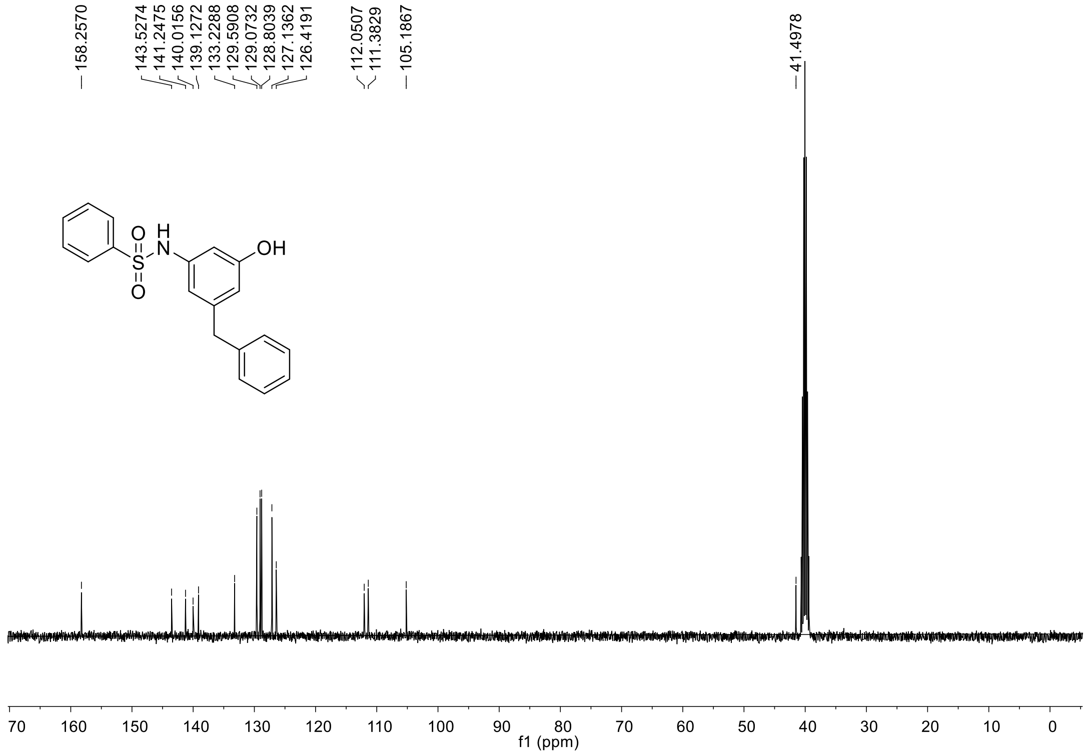
158.2570
143.5274
141.2475
140.0156
139.1272
133.2288
129.5908
129.0732
128.8039
127.1362
126.4191
112.0507
111.3829
105.1867
41.4978
OH
f1 (ppm)

Compound **1b**

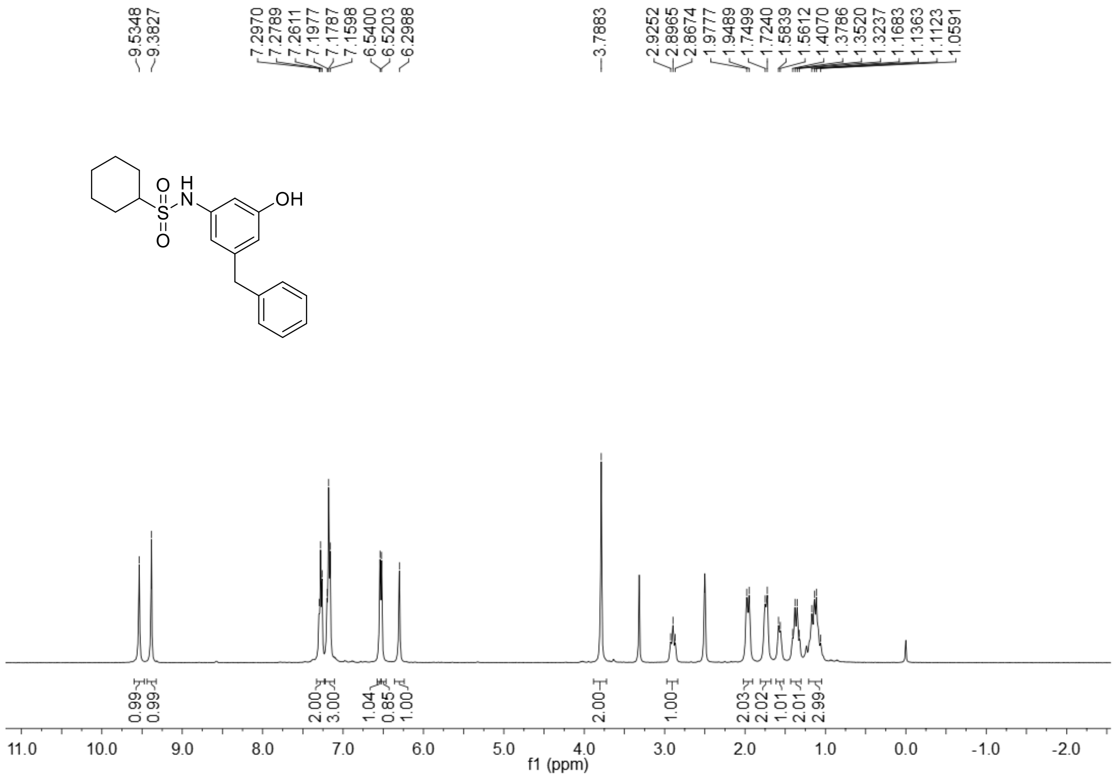
9.5348
9.3827
7.2970
7.2789
7.2611
7.1977
7.1787
7.1598
6.5400
6.5203
6.2988
3.7883
2.9252
2.8965
2.8674
1.9777
1.9489
1.7499
1.7240
1.5839
1.5612
1.4070
1.3786
1.3520
1.3237
1.1683
1.1363
1.1123
1.0591
OH
O
S
O
H
N
0.99
0.99
2.00
3.00
1.04
0.85
1.00
2.00
1.00
2.03
2.02
1.01
2.01
2.99
11.0
10.0
9.0
8.0
7.0
6.0
5.0
4.0
3.0
2.0
1.0
0.0
-1.0
-2.0
f1 (ppm)

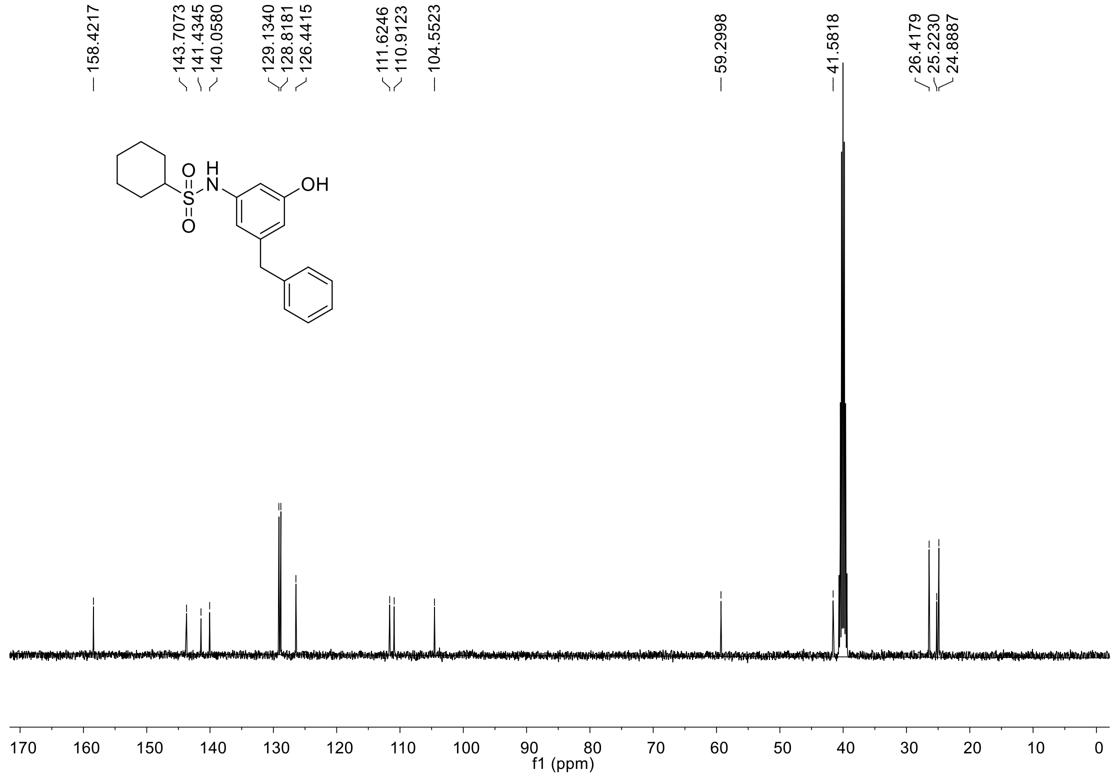
158.4217
143.7073
141.4345
140.0580
129.1340
128.8181
126.4415
111.6246
110.9123
104.5523
59.2998
41.5818
26.4179
25.2230
24.8887
OH
O
S
O
H
N
170
160
150
140
130
120
110
100
90
80
70
60
50
40
30
20
10
0
f1 (ppm)

Compound **2a**

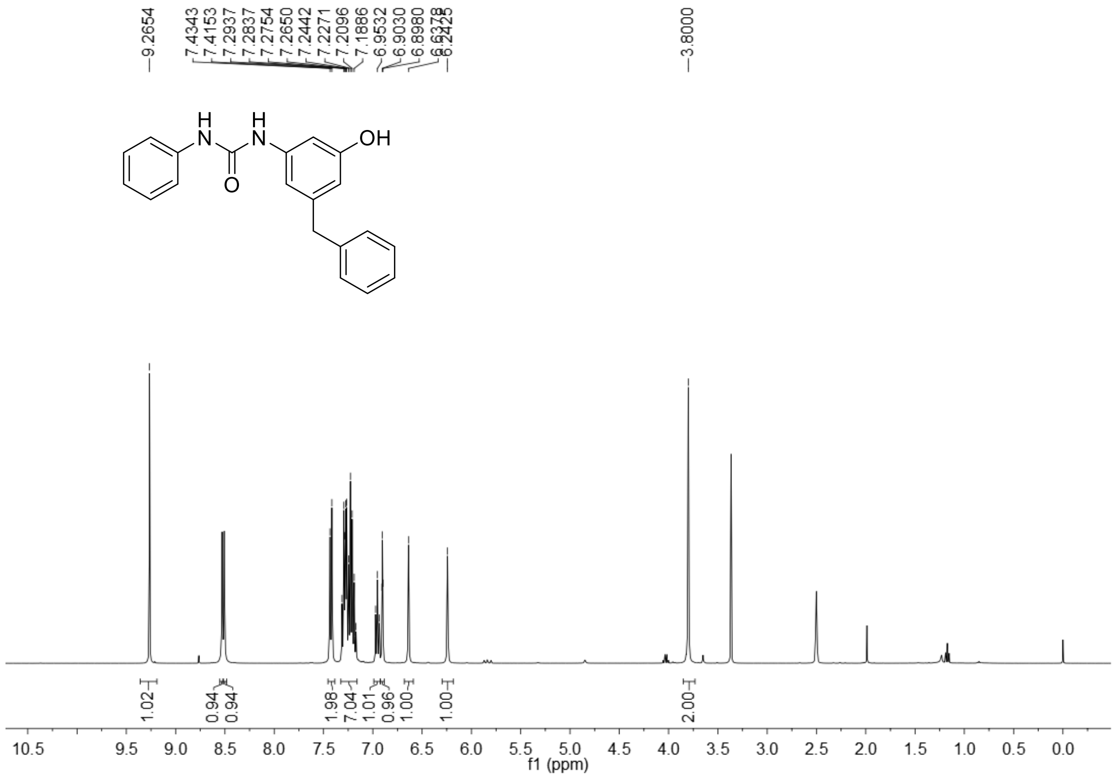

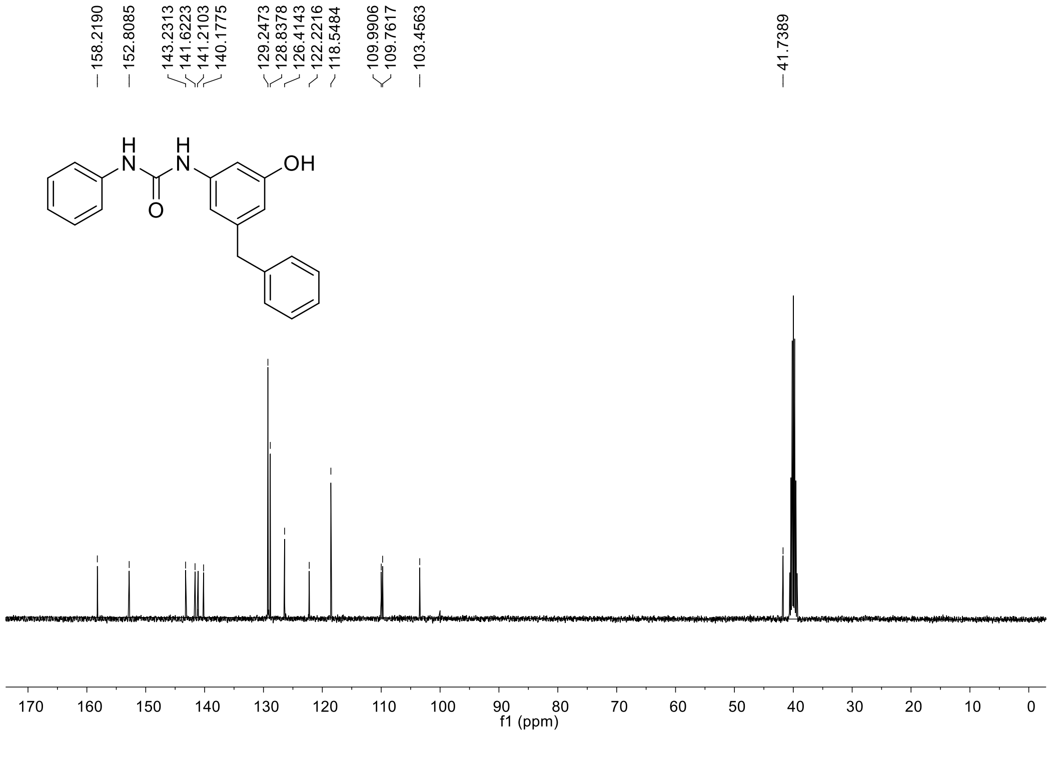

Compound **2b**

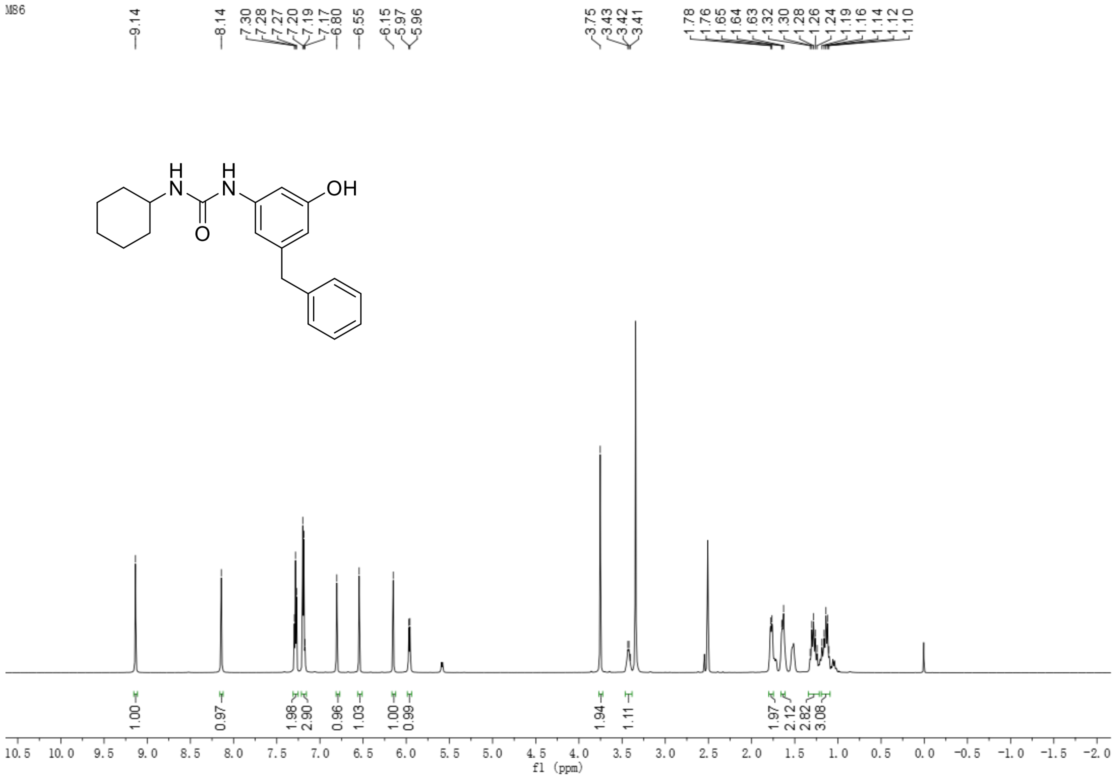
MS6
9.14
8.14
7.30
7.28
7.27
7.20
7.19
7.17
6.80
6.55
6.15
5.97
5.96
3.75
3.43
3.42
3.41
1.78
1.76
1.65
1.64
1.63
1.32
1.30
1.28
1.26
1.24
1.19
1.16
1.14
1.12
1.10
H
N
H
N
OH
O
1.00
0.97
1.98
2.90
0.96
1.03
1.00
0.99
1.94
1.11
1.97
2.12
2.82
3.08
10.5 10.0 9.5 9.0 8.5 8.0 7.5 7.0 6.5 6.0 5.5 5.0 4.5 4.0 3.5 3.0 2.5 2.0 1.5 1.0 0.5 0.0 -0.5 -1.0 -1.5 -2.0
f1 (ppm)

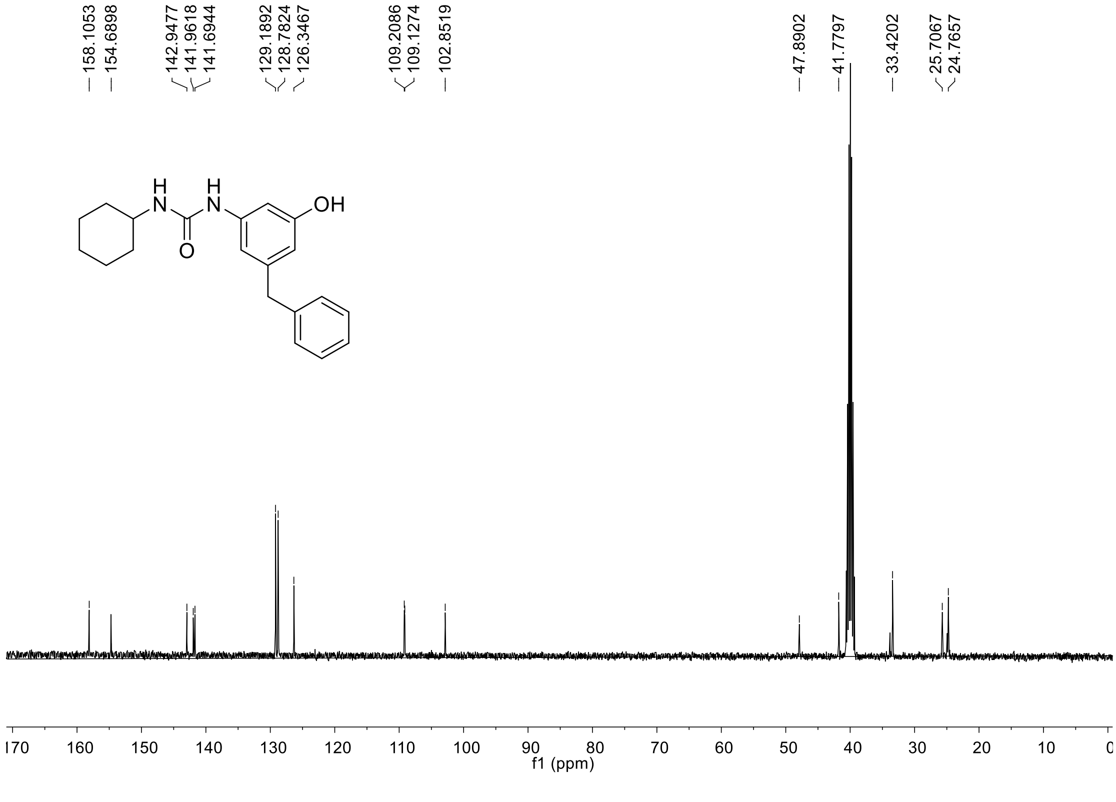
158.1053
154.6898
142.9477
141.9618
141.6944
129.1892
128.7824
126.3467
109.2086
109.1274
102.8519
47.8902
41.7797
33.4202
25.7067
24.7657
H
N
H
N
OH
O
170 160 150 140 130 120 110 100 90 80 70 60 50 40 30 20 10 0
f1 (ppm)

Compound **3a**

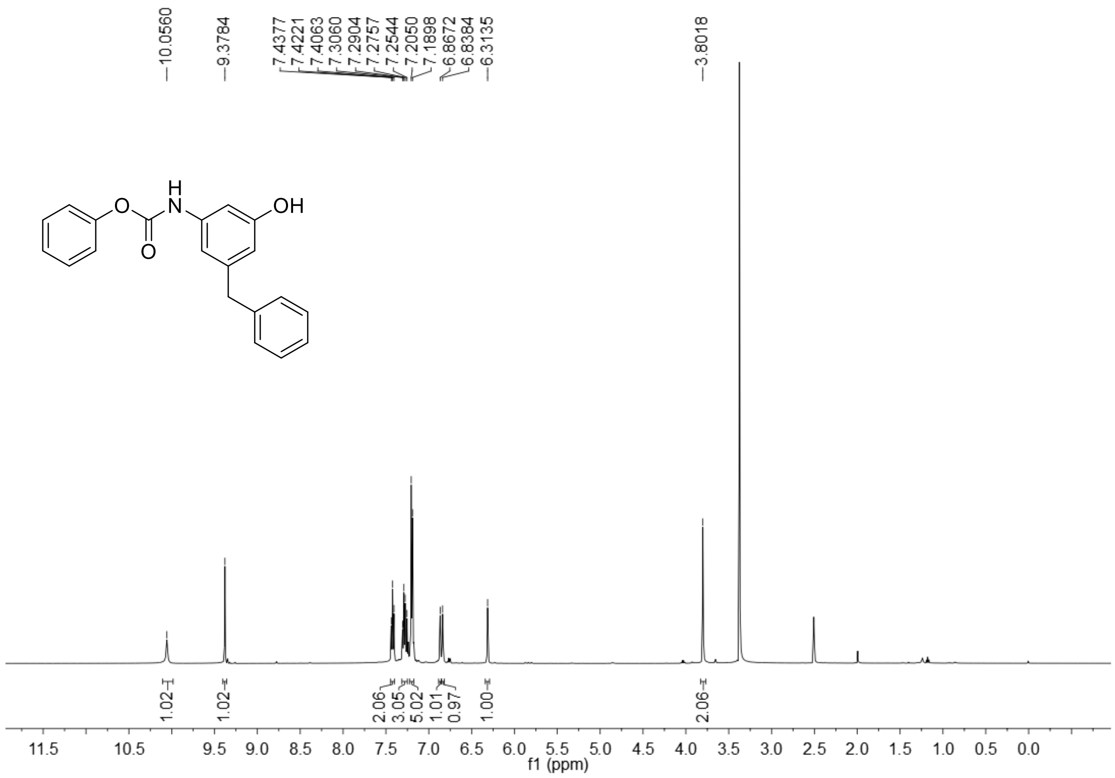

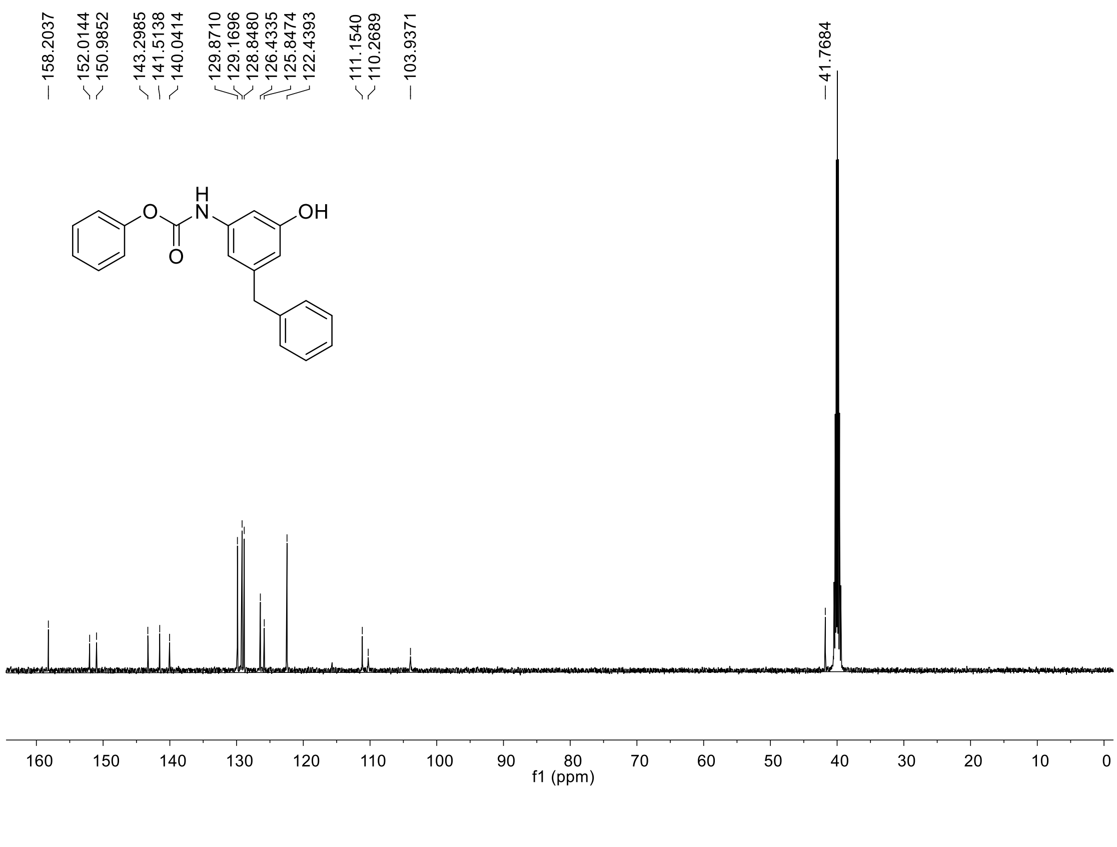

Compound **3b**

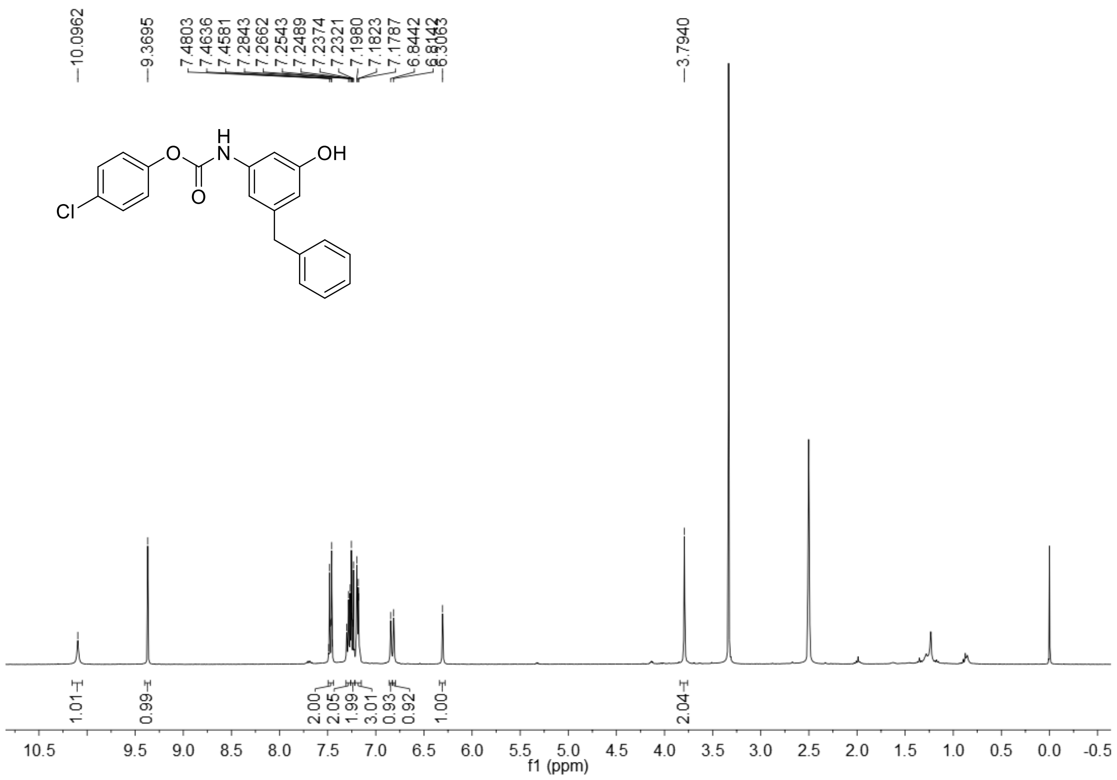

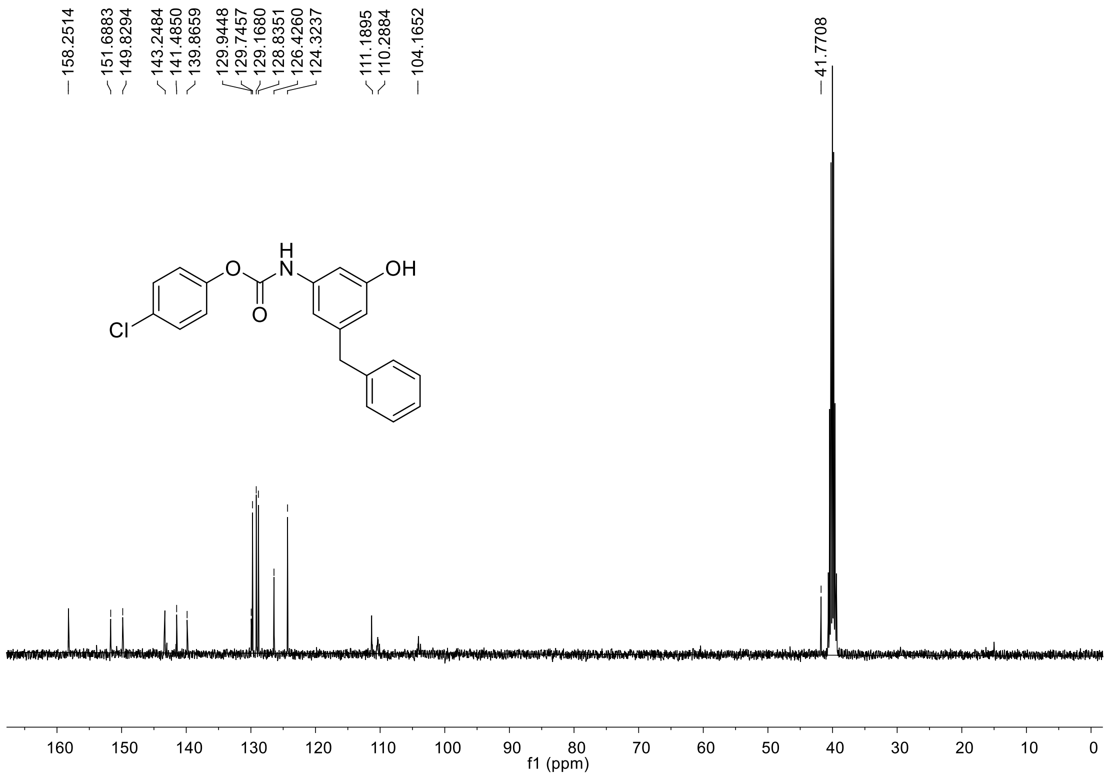

Compound **3c**

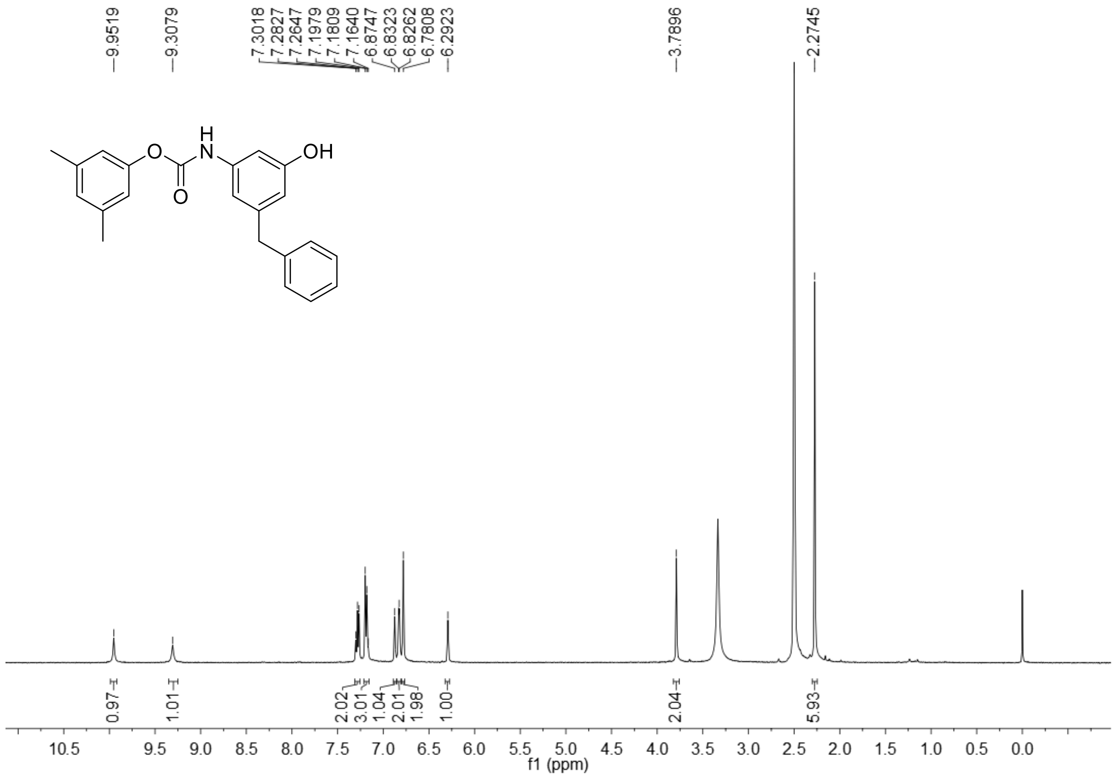
9.9519
9.3079
7.3018
7.2827
7.2647
7.1979
7.1809
7.1640
6.8747
6.8323
6.8262
6.7808
6.2923
3.7896
2.2745
0.97
1.01
2.02
3.01
1.04
2.01
1.98
1.00
2.04
5.93
f1 (ppm)

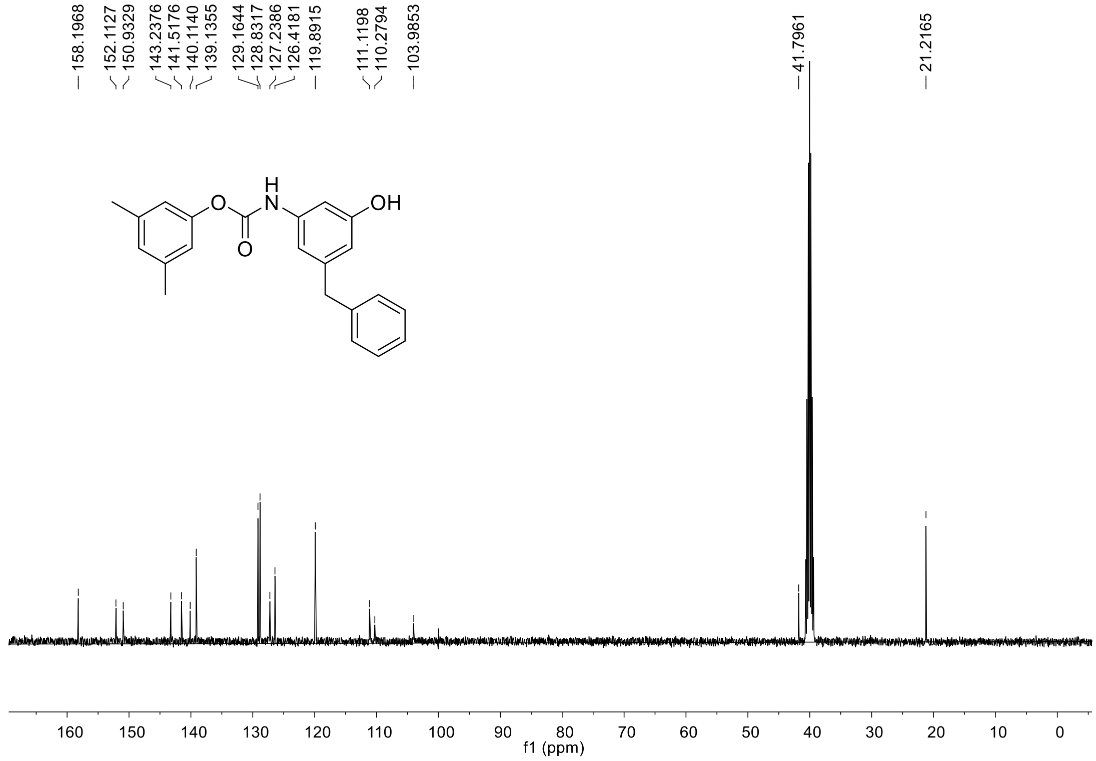
158.1968
152.1127
150.9329
143.2376
141.5176
140.1140
139.1355
129.1644
128.8317
127.2386
126.4181
119.8915
111.1198
110.2794
103.9853
41.7961
21.2165
f1 (ppm)

Compound **3d**

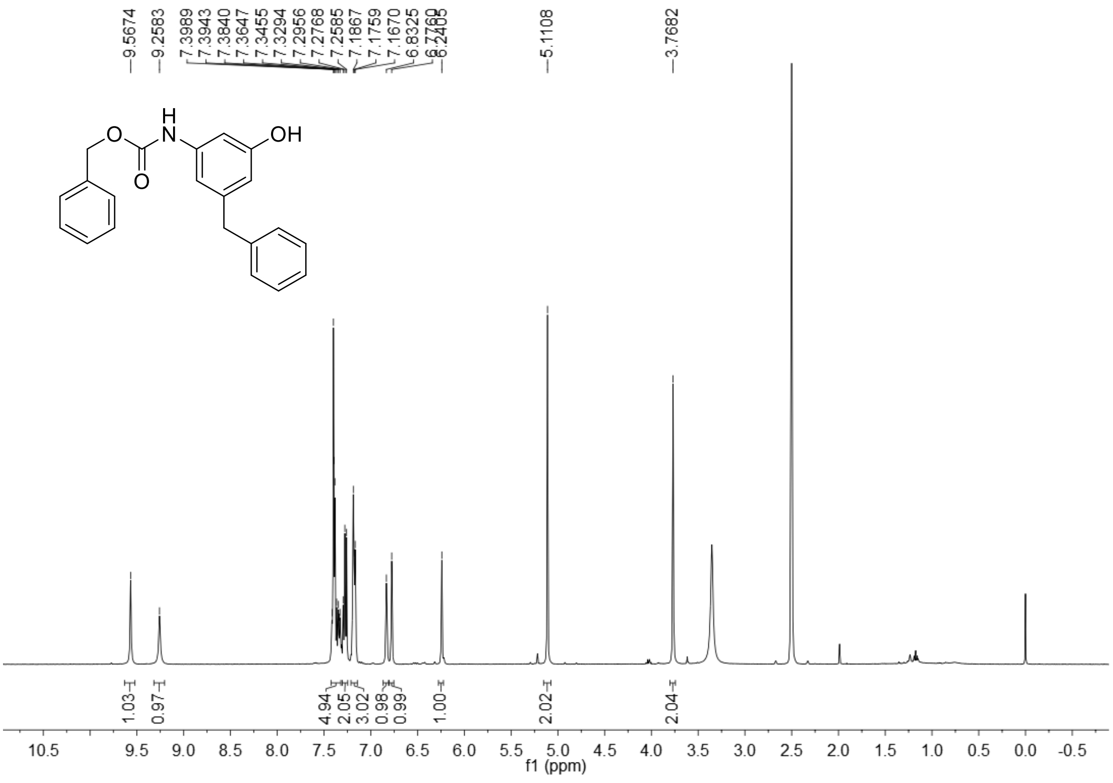

9.5674
9.2583
7.3989
7.3943
7.3840
7.3647
7.3455
7.3294
7.2956
7.2768
7.2585
7.1867
7.1759
7.1670
6.8325
5.1108
3.7682
OH
H
N
O
1.03
0.97
4.94
2.05
3.02
0.98
0.99
1.00
2.02
2.04
10.5
9.5
9.0
8.5
8.0
7.5
7.0
6.5
6.0
5.5
5.0
4.5
4.0
3.5
3.0
2.5
2.0
1.5
1.0
0.5
0.0
-0.5
f1 (ppm)

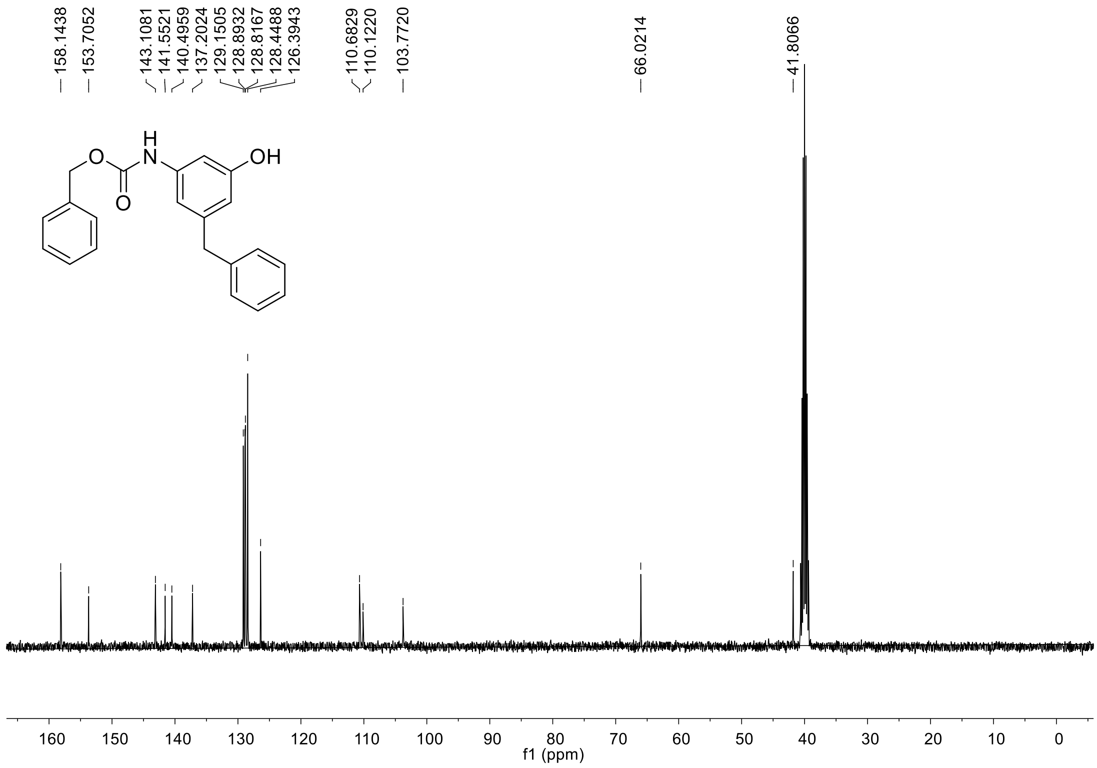

158.1438
153.7052
143.1081
141.5521
140.4959
137.2024
129.1505
128.8932
128.8167
128.4488
126.3943
110.6829
110.1220
103.7720
66.0214
41.8066
OH
H
N
O
160
150
140
130
120
110
100
90
80
70
60
50
40
30
20
10
0
f1 (ppm)

Compound **3e**

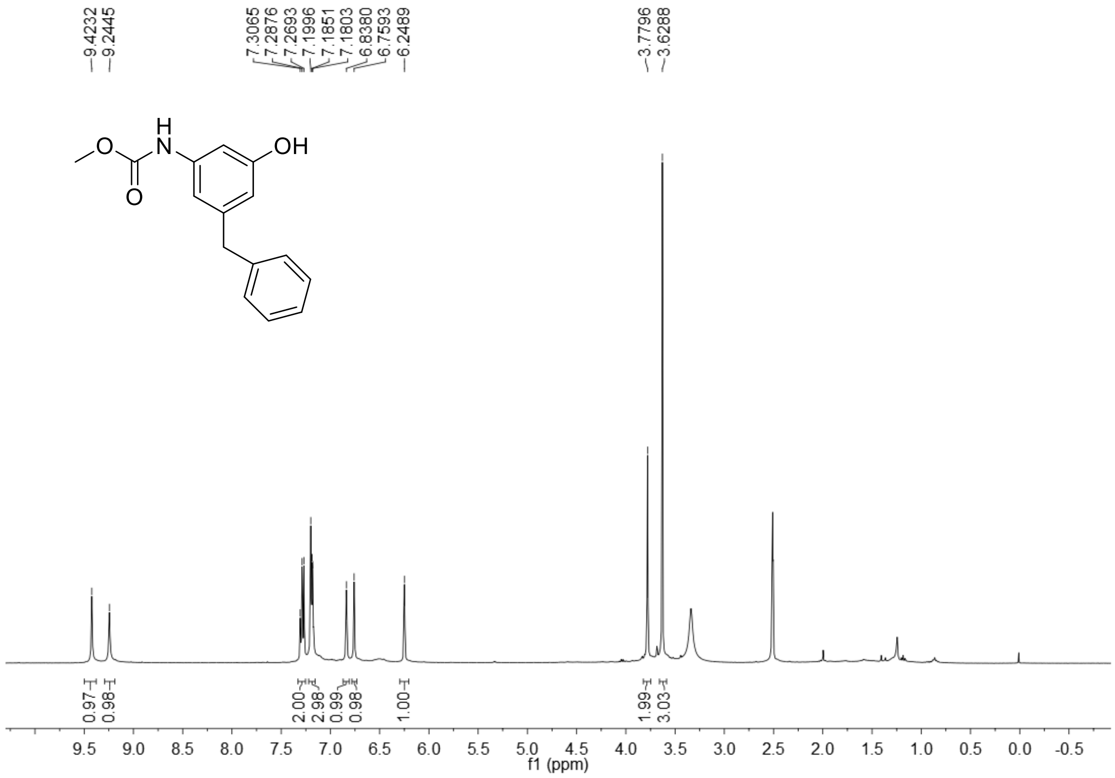
9.4232
9.2445
7.3065
7.2876
7.2693
7.1996
7.1851
7.1803
6.8380
6.7593
6.2489
3.7796
3.6288
H
N
O
O
OH
0.97
0.98
2.00
2.98
0.99
0.98
1.00
1.99
3.03
9.5 9.0 8.5 8.0 7.5 7.0 6.5 6.0 5.5 5.0 4.5 4.0 3.5 3.0 2.5 2.0 1.5 1.0 0.5 0.0 -0.5
f1 (ppm)

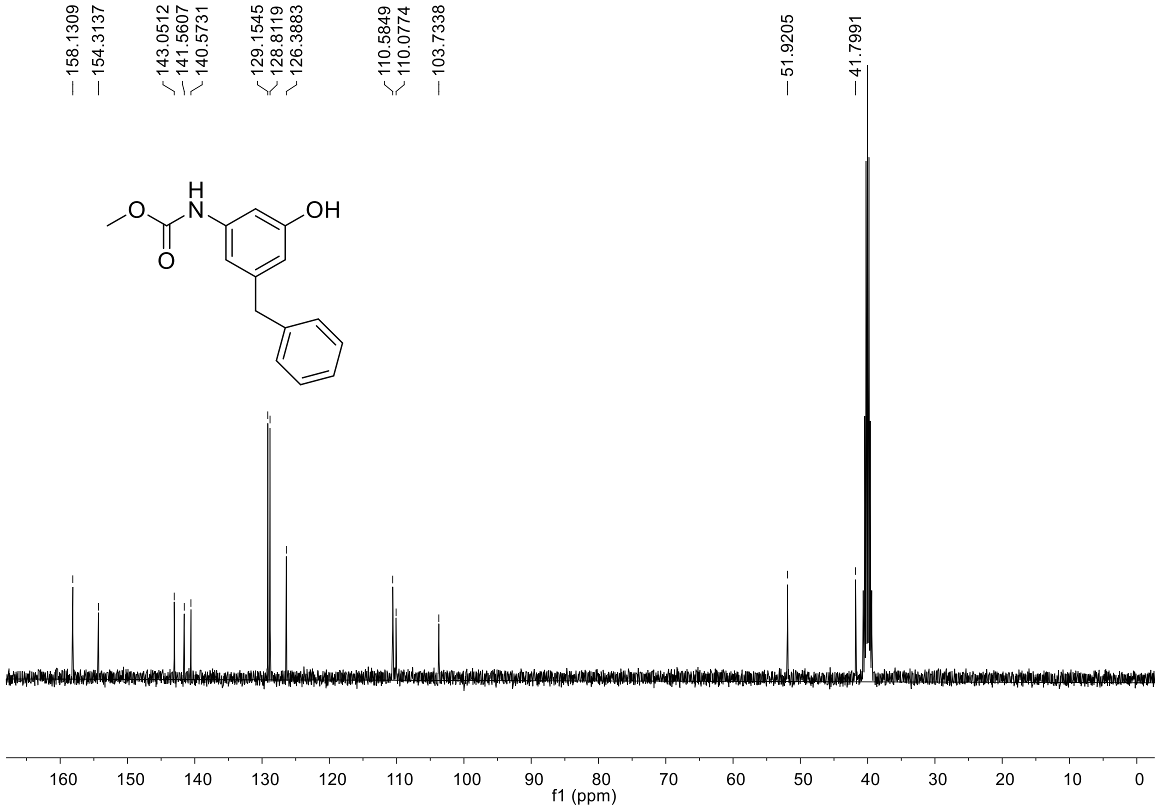
158.1309
154.3137
143.0512
141.5607
140.5731
129.1545
128.8119
126.3883
110.5849
110.0774
103.7338
51.9205
41.7991
H
N
O
O
OH
160 150 140 130 120 110 100 90 80 70 60 50 40 30 20 10 0
f1 (ppm)

Compound **3f**

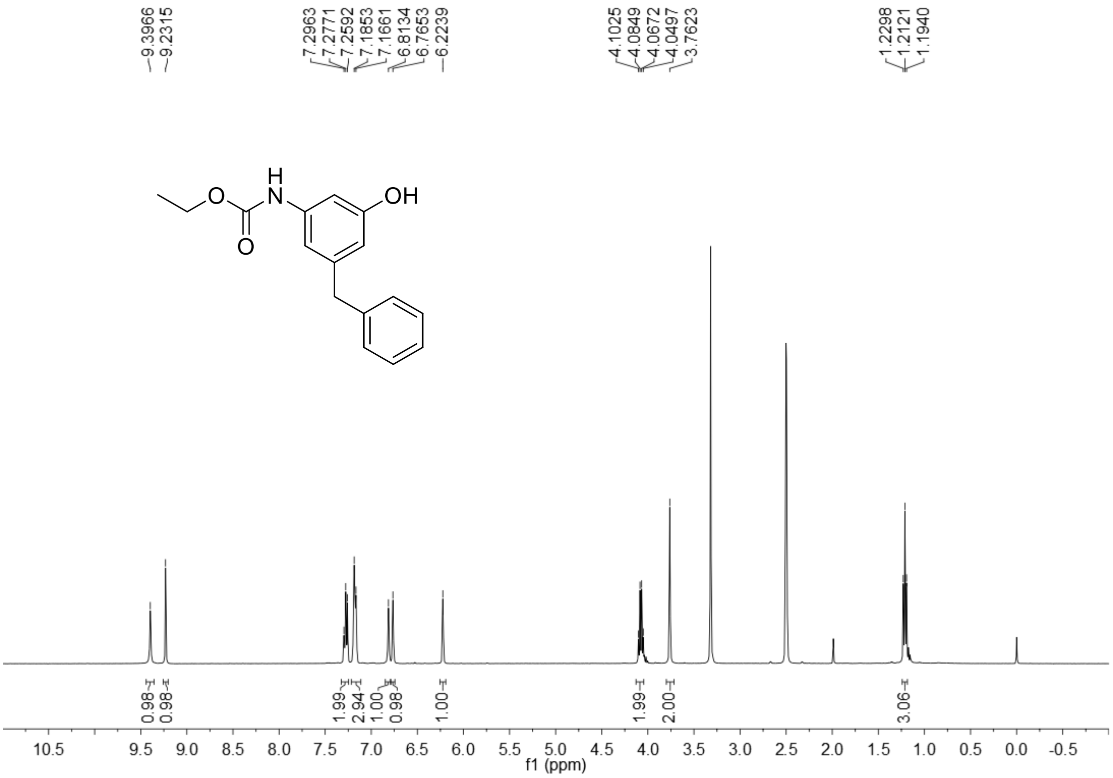
9.3966
9.2315
7.2963
7.2771
7.2592
7.1853
7.1661
6.8134
6.7653
6.2239
4.1025
4.0849
4.0672
4.0497
3.7623
1.2298
1.2121
1.1940
H
N
O
O
OH
0.98
0.98
1.99
2.94
1.00
0.98
1.00
1.99
2.00
3.06
10.5 9.5 9.0 8.5 8.0 7.5 7.0 6.5 6.0 5.5 5.0 4.5 4.0 3.5 3.0 2.5 2.0 1.5 1.0 0.5 0.0 -0.5
f1 (ppm)

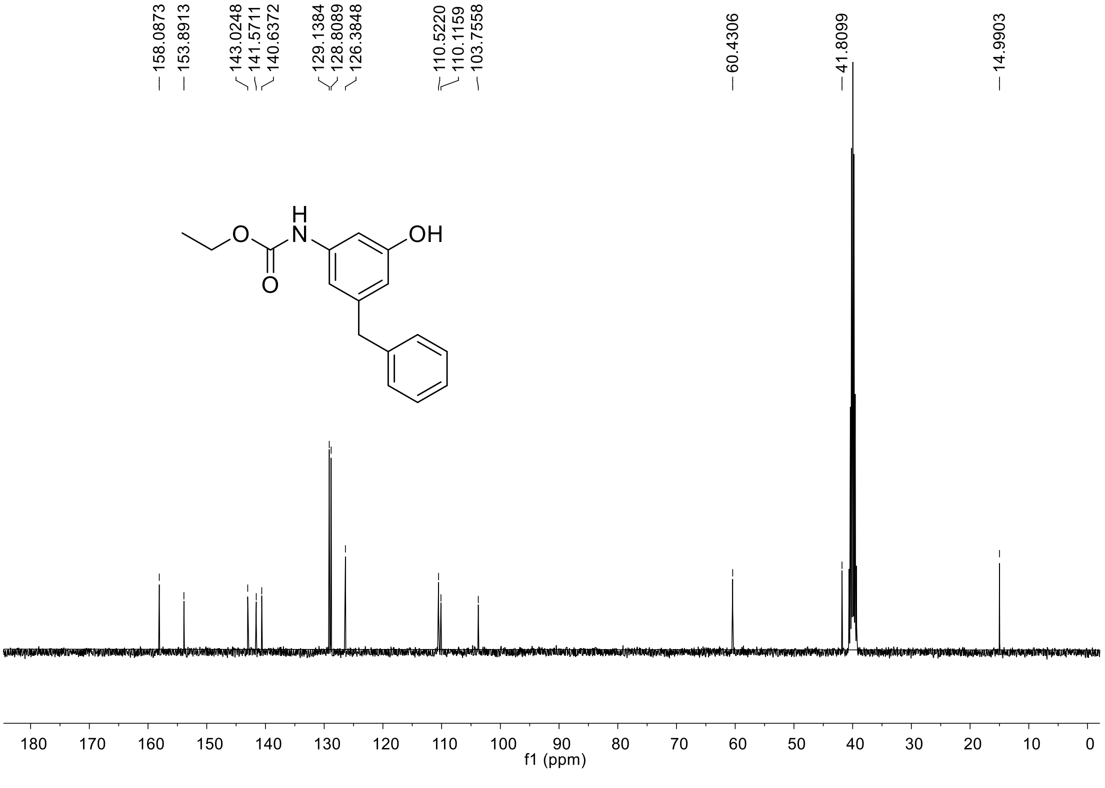
158.0873
153.8913
143.0248
141.5711
140.6372
129.1384
128.8089
126.3848
110.5220
110.1159
103.7558
60.4306
41.8099
14.9903
H
N
O
O
OH
180 170 160 150 140 130 120 110 100 90 80 70 60 50 40 30 20 10 0
f1 (ppm)

Compound **3g**

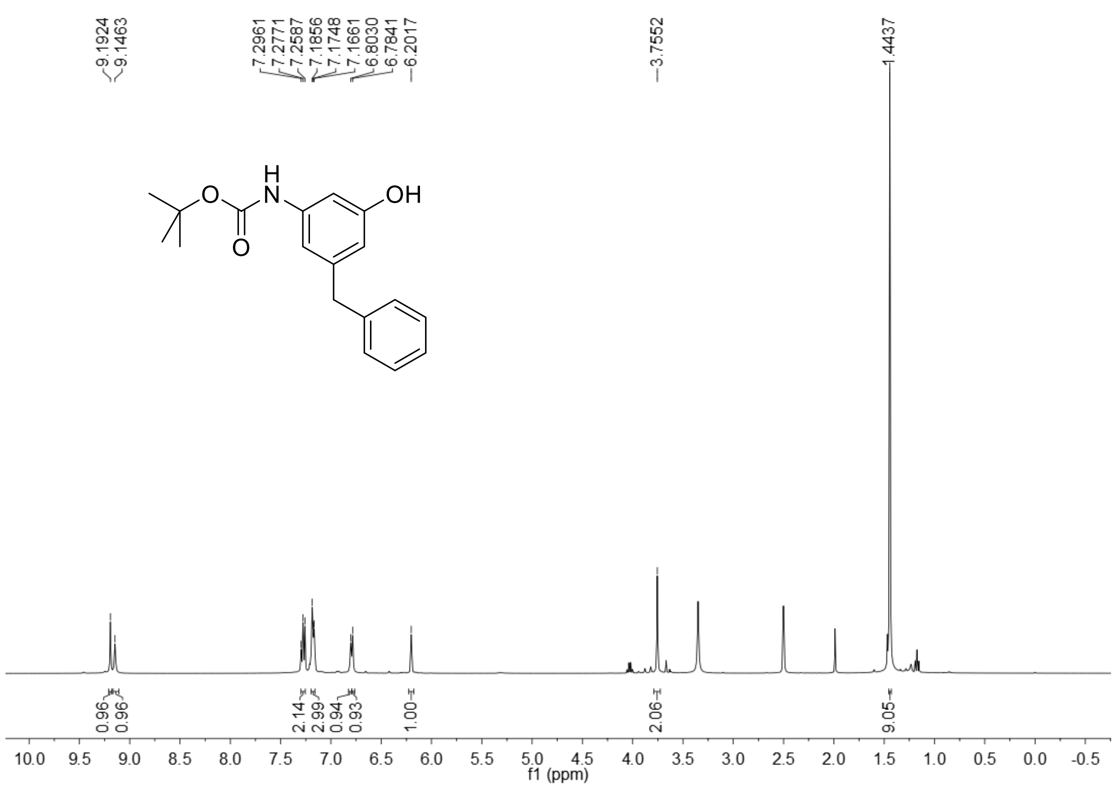
9.1924
9.1463
7.2961
7.2771
7.2587
7.1856
7.1748
7.1661
6.8030
6.7841
6.2017
3.7552
1.4437
OH
0.96
0.96
2.14
2.99
0.94
0.93
1.00
2.06
9.05
f1 (ppm)

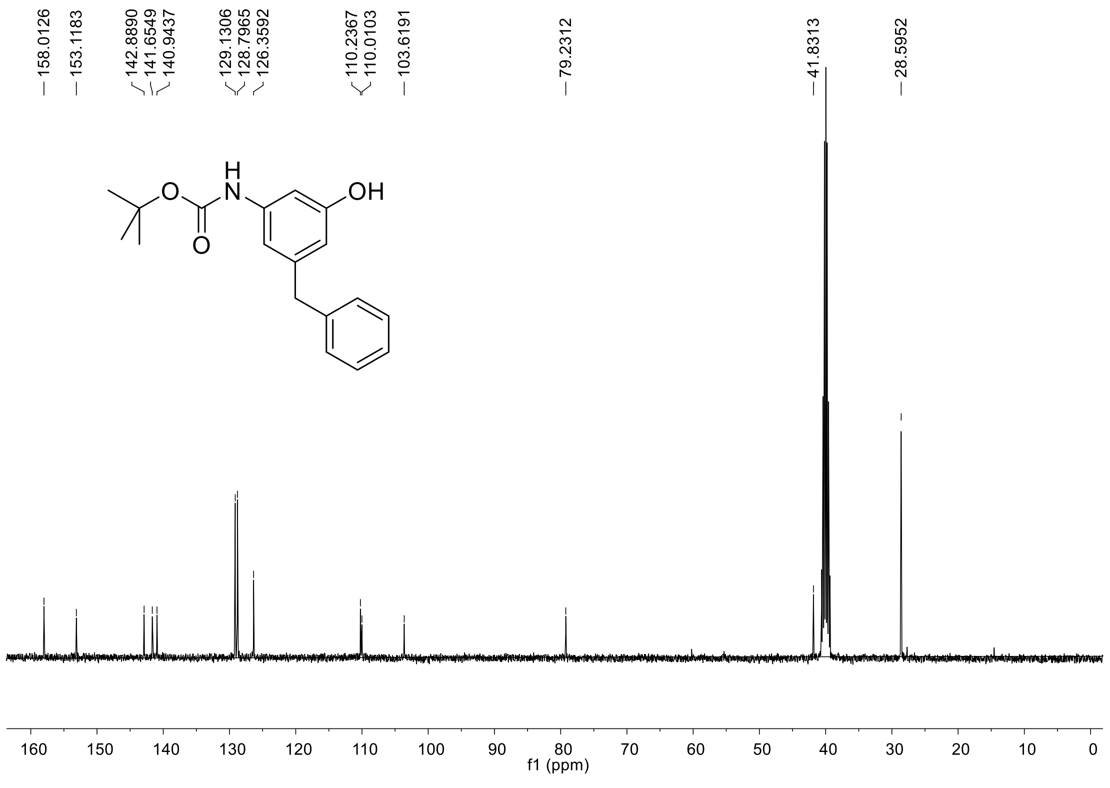
158.0126
153.1183
142.8890
141.6549
140.9437
129.1306
128.7965
126.3592
110.2367
110.0103
103.6191
79.2312
41.8313
28.5952
OH
f1 (ppm)

Compound **3h**

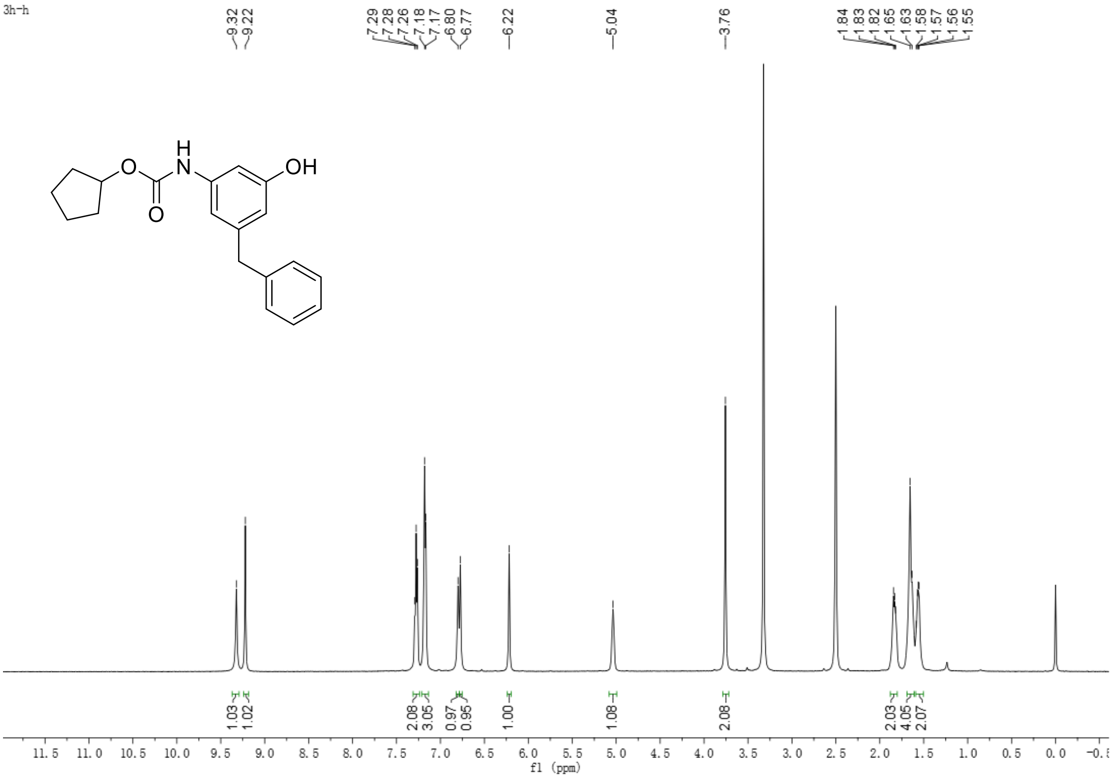
3h-h
9.32
9.22
7.29
7.28
7.26
7.18
7.17
6.80
6.77
6.22
5.04
3.76
1.84
1.83
1.82
1.65
1.63
1.58
1.57
1.56
1.55
1.03
1.02
2.08
3.05
0.97
0.95
1.00
1.08
2.08
2.03
4.05
2.07
f1 (ppm)

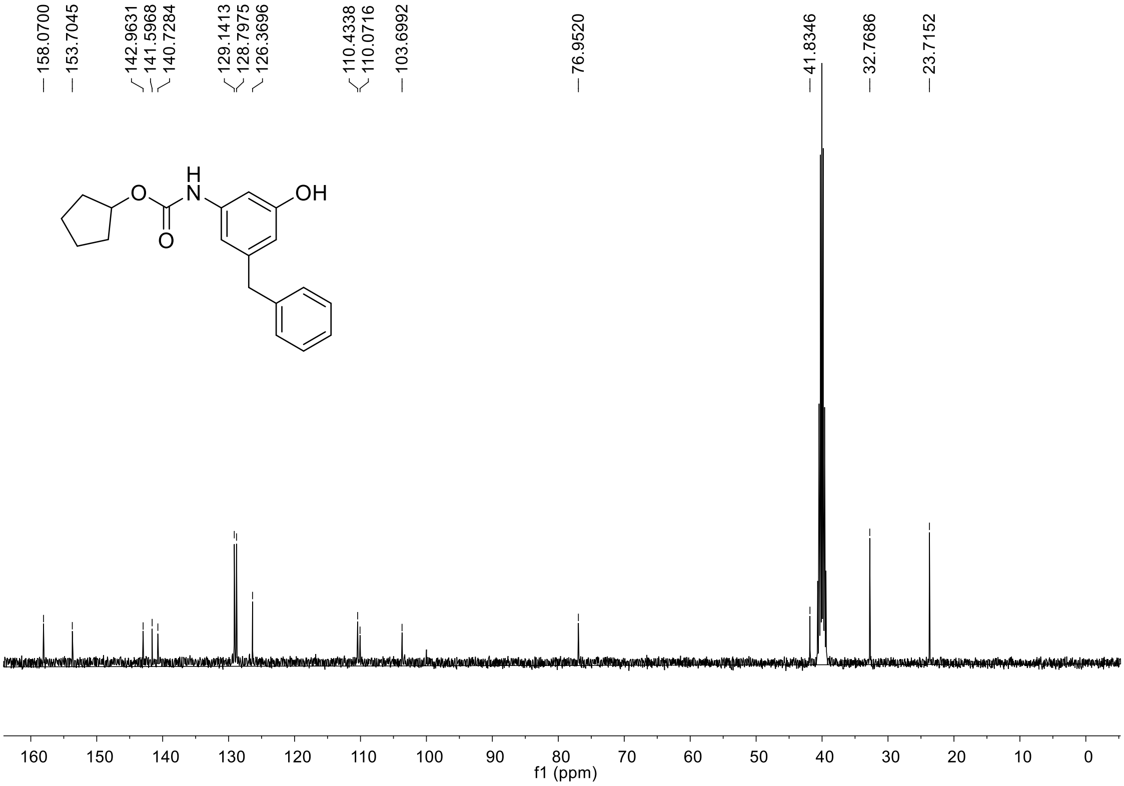
158.0700
153.7045
142.9631
141.5968
140.7284
129.1413
128.7975
126.3696
110.4338
110.0716
103.6992
76.9520
41.8346
32.7686
23.7152
f1 (ppm)

Compound **3i**

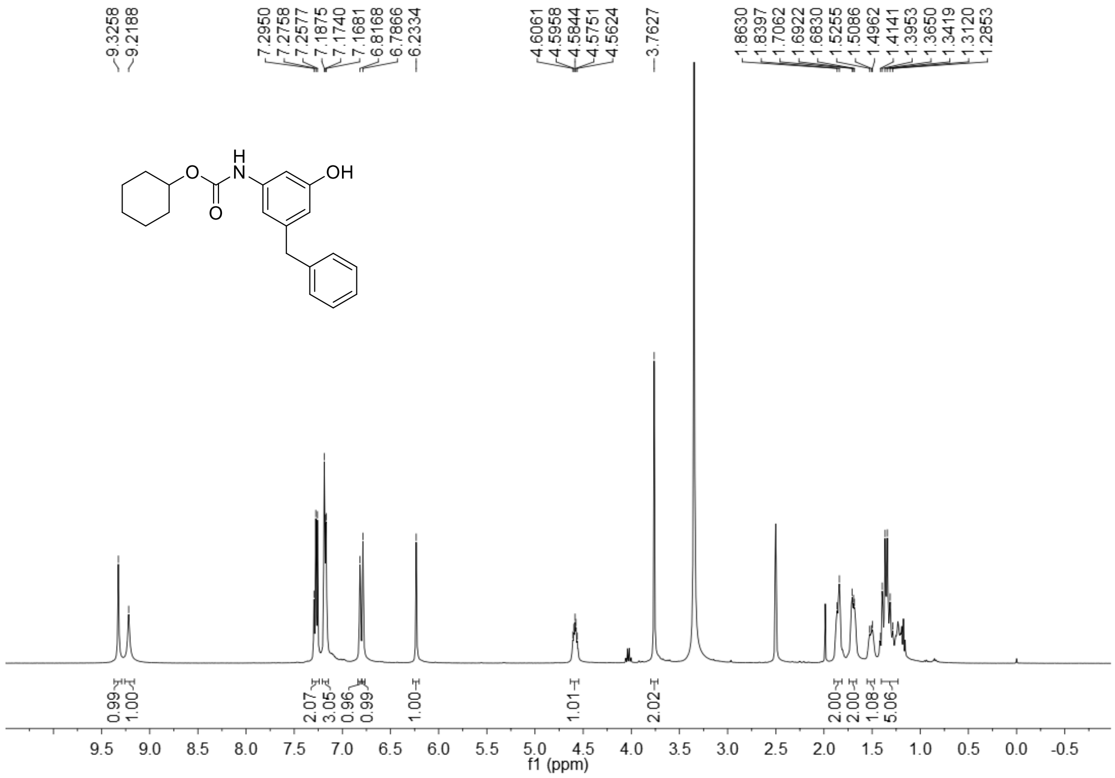
9.3258
9.2188
7.2950
7.2758
7.2577
7.1875
7.1740
7.1681
6.8168
6.7866
6.2334
4.6061
4.5958
4.5844
4.5751
4.5624
3.7627
1.8630
1.8397
1.7062
1.6922
1.6830
1.5255
1.5086
1.4962
1.4141
1.3953
1.3650
1.3419
1.3120
1.2853
OH
H
N
O
O
0.99
1.00
2.07
3.05
0.96
0.99
1.00
1.01
2.02
2.00
2.00
1.08
5.06
f1 (ppm)

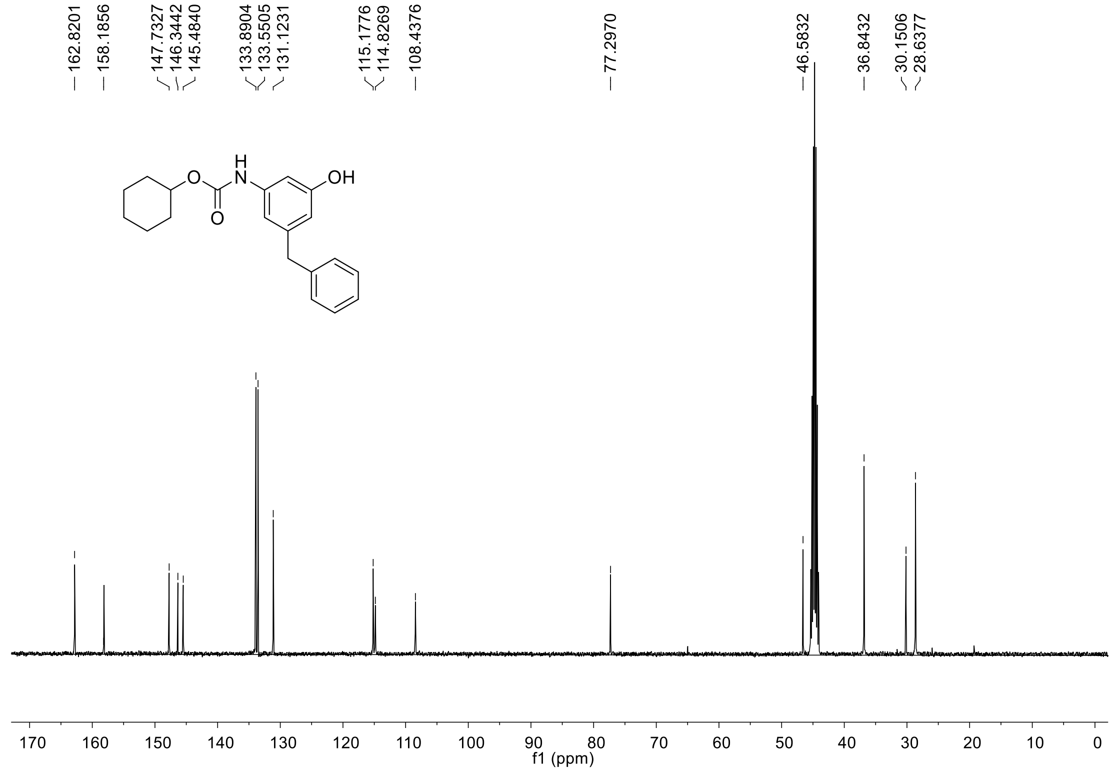
162.8201
158.1856
147.7327
146.3442
145.4840
133.8904
133.5505
131.1231
115.1776
114.8269
108.4376
77.2970
46.5832
36.8432
30.1506
28.6377
OH
H
N
O
O
f1 (ppm)

Compound **3j**

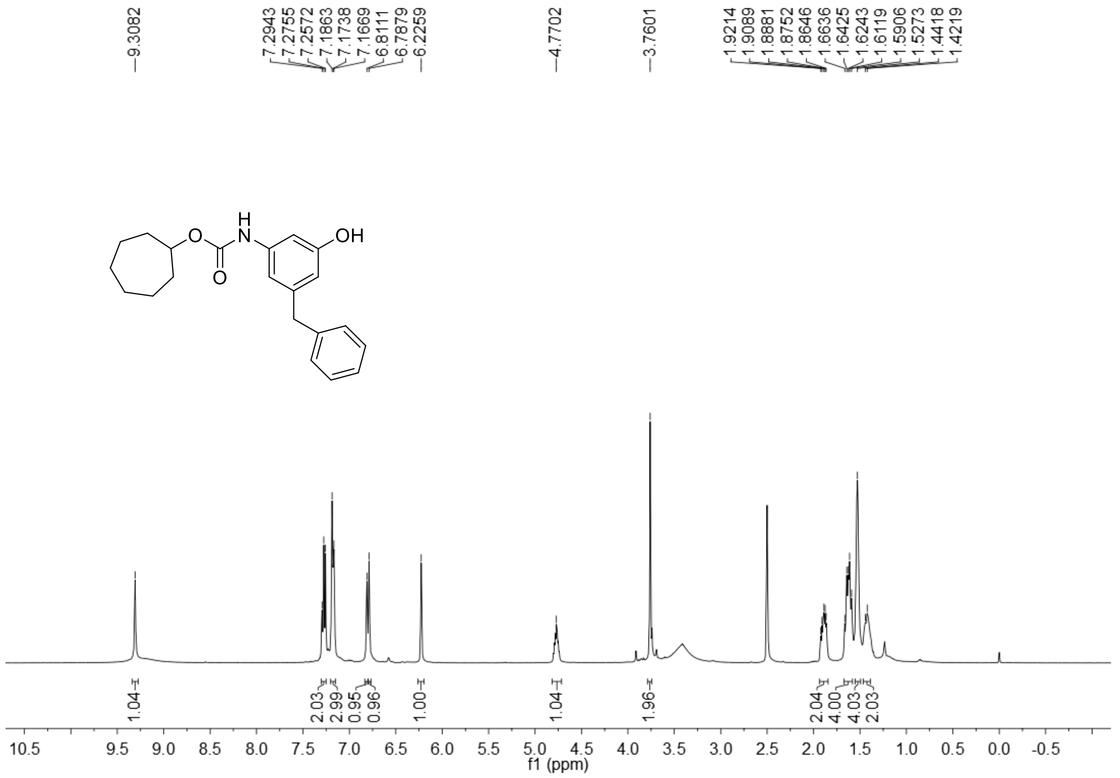
9.3082
7.2943
7.2755
7.2572
7.1863
7.1738
7.1669
6.8111
6.7879
6.2259
4.7702
3.7601
1.9214
1.9089
1.8881
1.8752
1.8646
1.6636
1.6425
1.6243
1.6119
1.5906
1.5273
1.4418
1.4219
H
N
O
O
OH
1.04
2.03
2.99
0.95
0.96
1.00
1.04
1.96
2.04
4.00
4.03
2.03
10.5
9.5
9.0
8.5
8.0
7.5
7.0
6.5
6.0
5.5
5.0
4.5
4.0
3.5
3.0
2.5
2.0
1.5
1.0
0.5
0.0
-0.5
f1 (ppm)

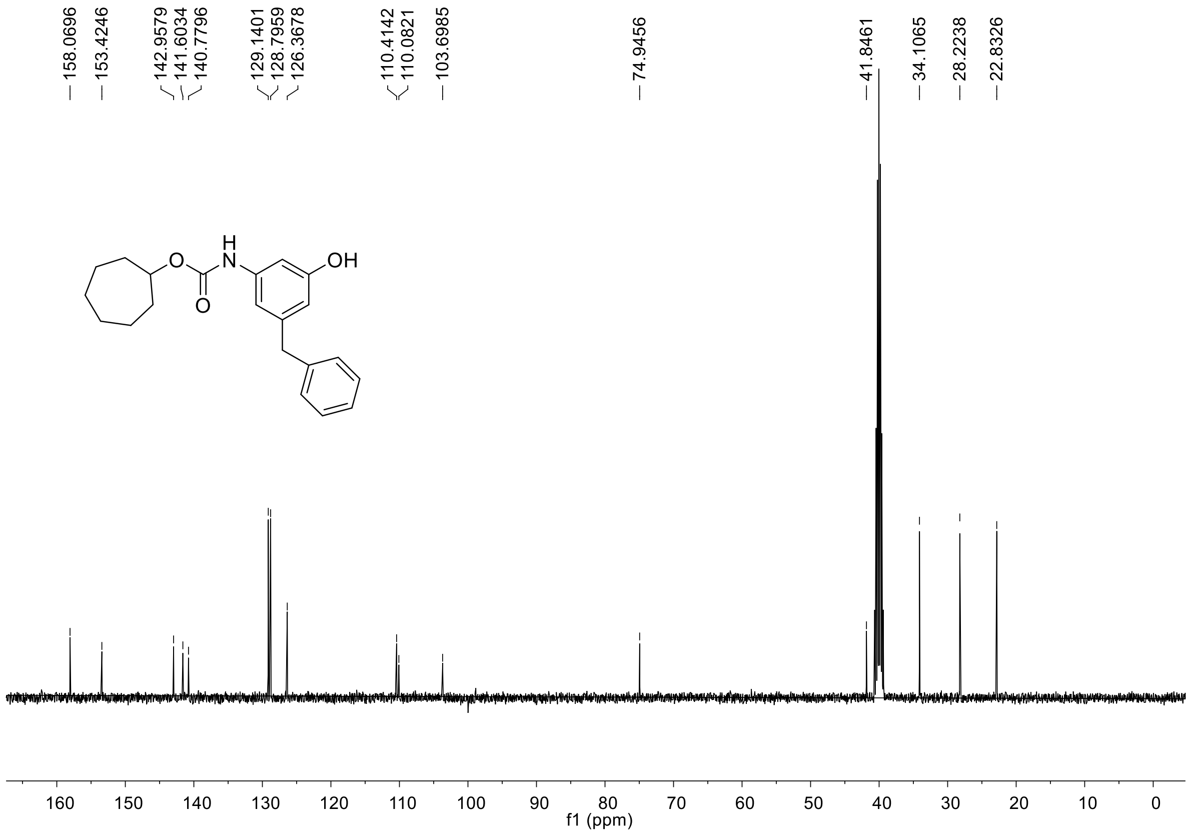
158.0696
153.4246
142.9579
141.6034
140.7796
129.1401
128.7959
126.3678
110.4142
110.0821
103.6985
74.9456
41.8461
34.1065
28.2238
22.8326
H
N
O
O
OH
160
150
140
130
120
110
100
90
80
70
60
50
40
30
20
10
0
f1 (ppm)

Compound **3k**

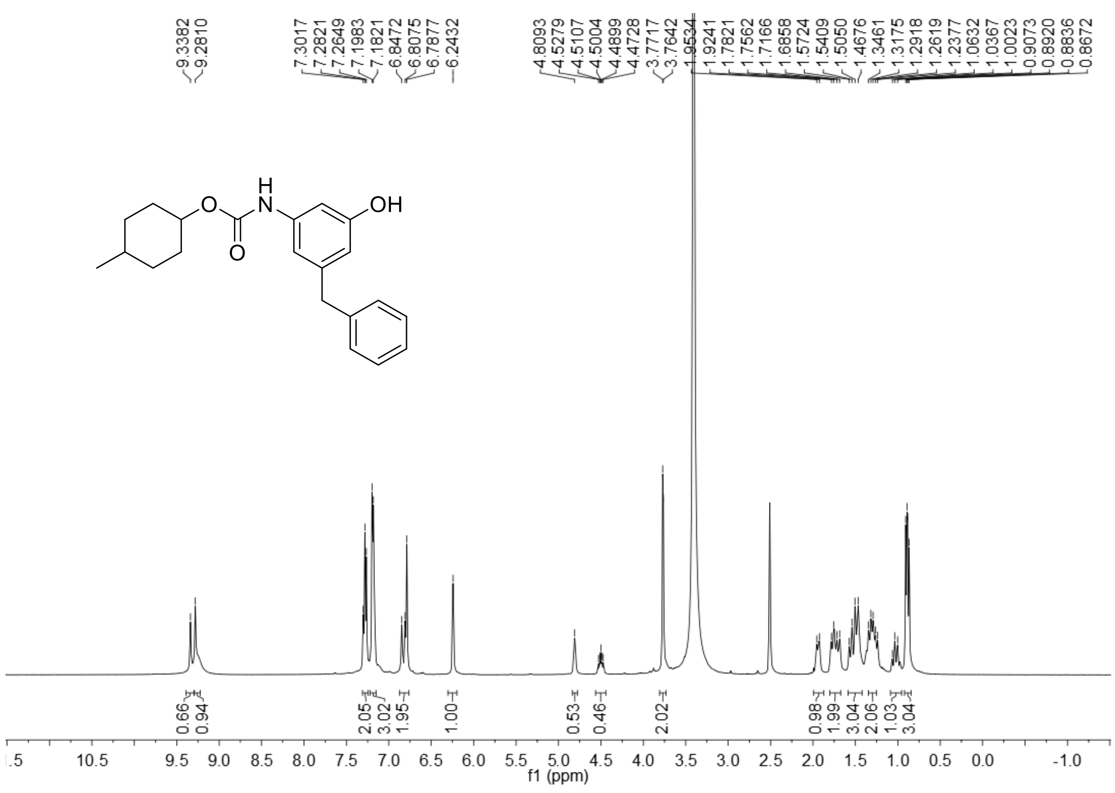

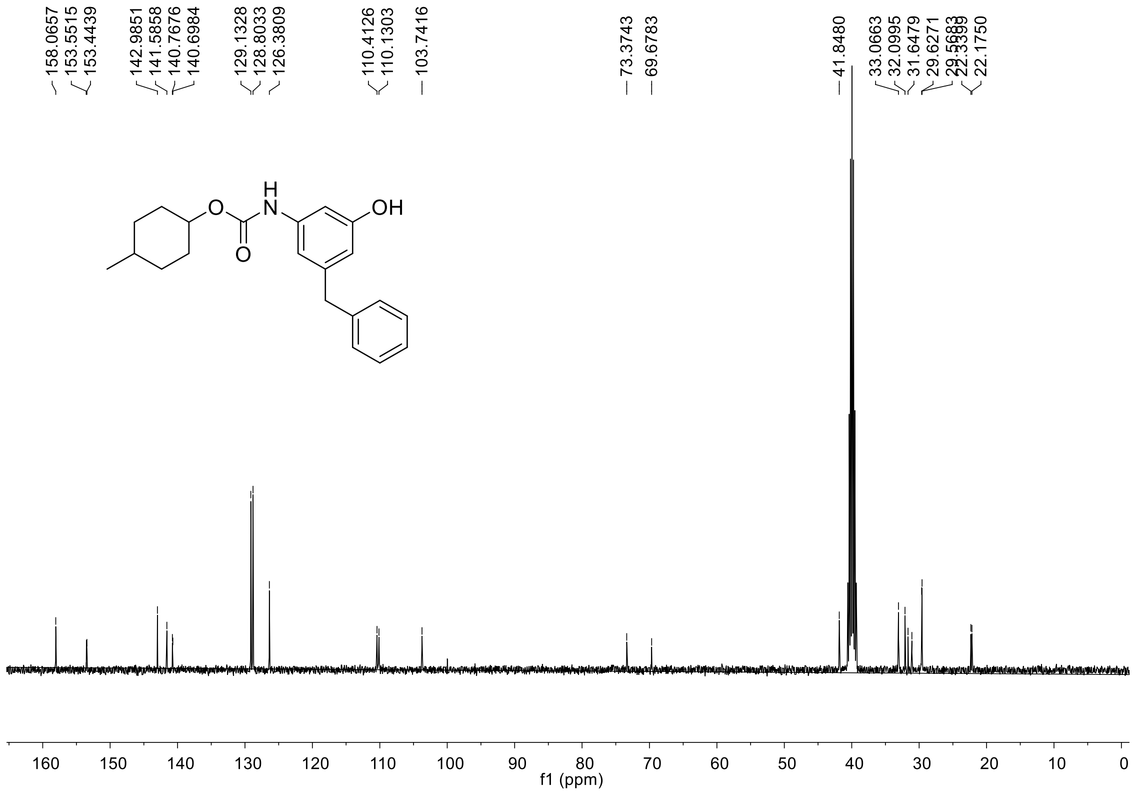

Compound **3l**

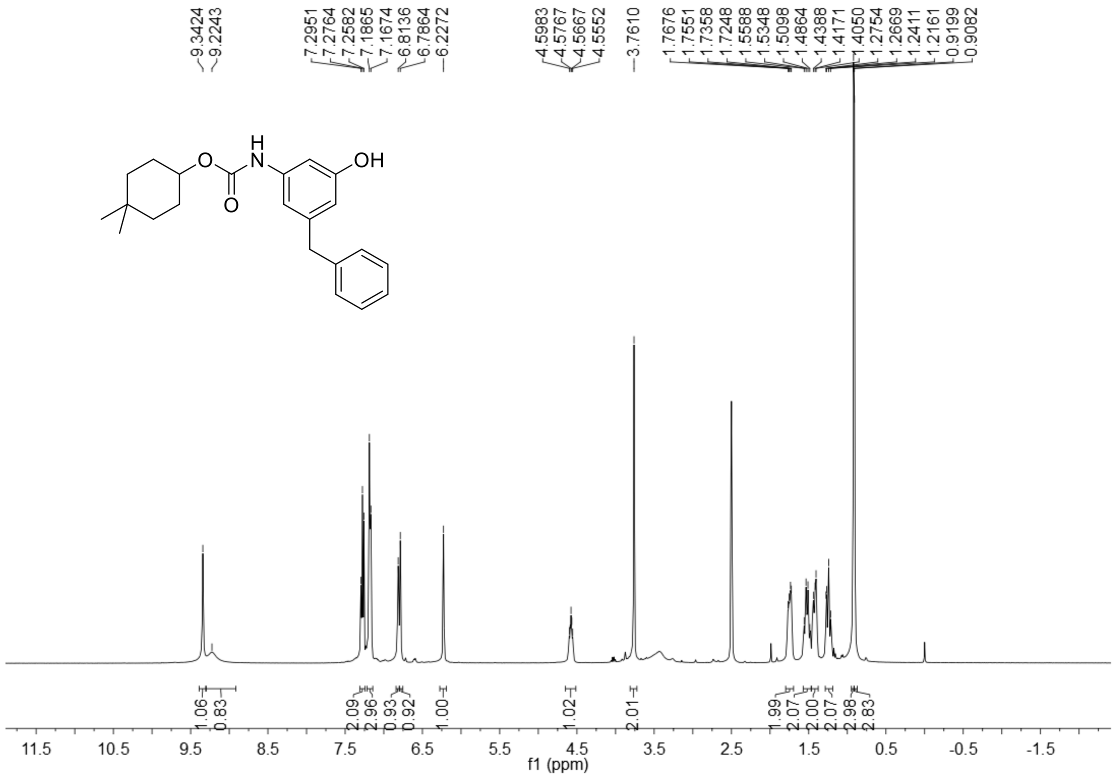

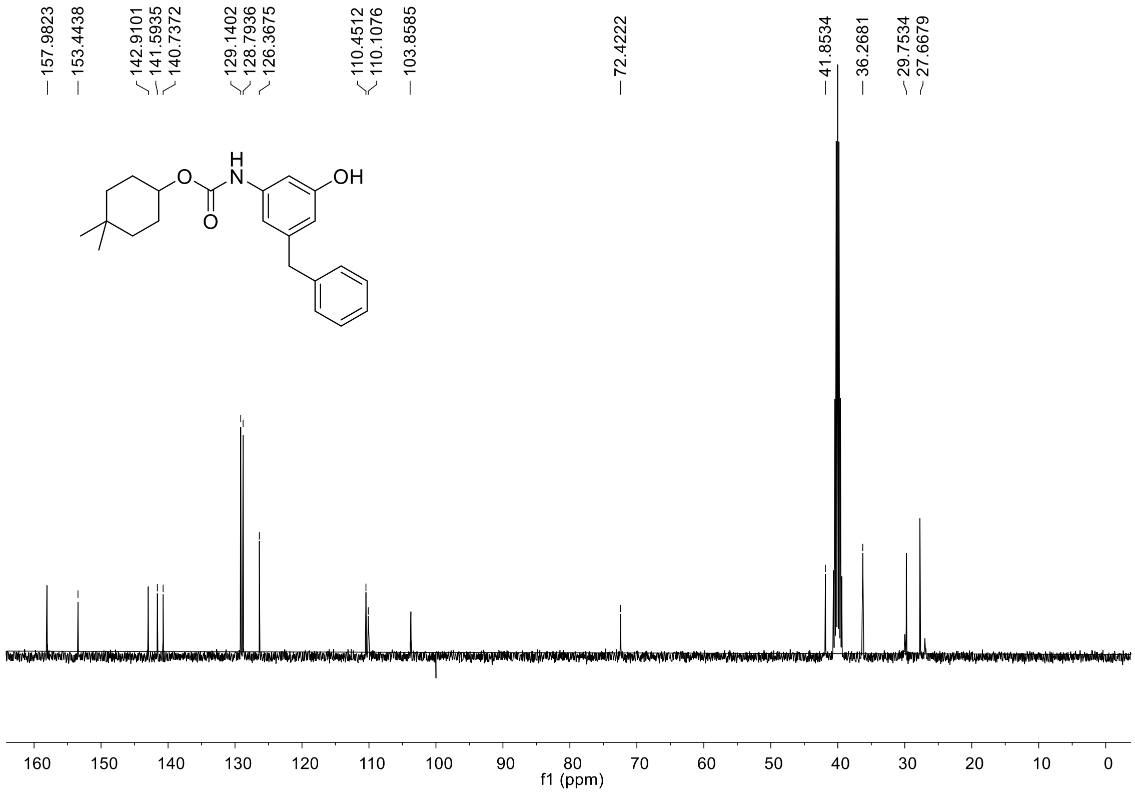

Compound **3m**

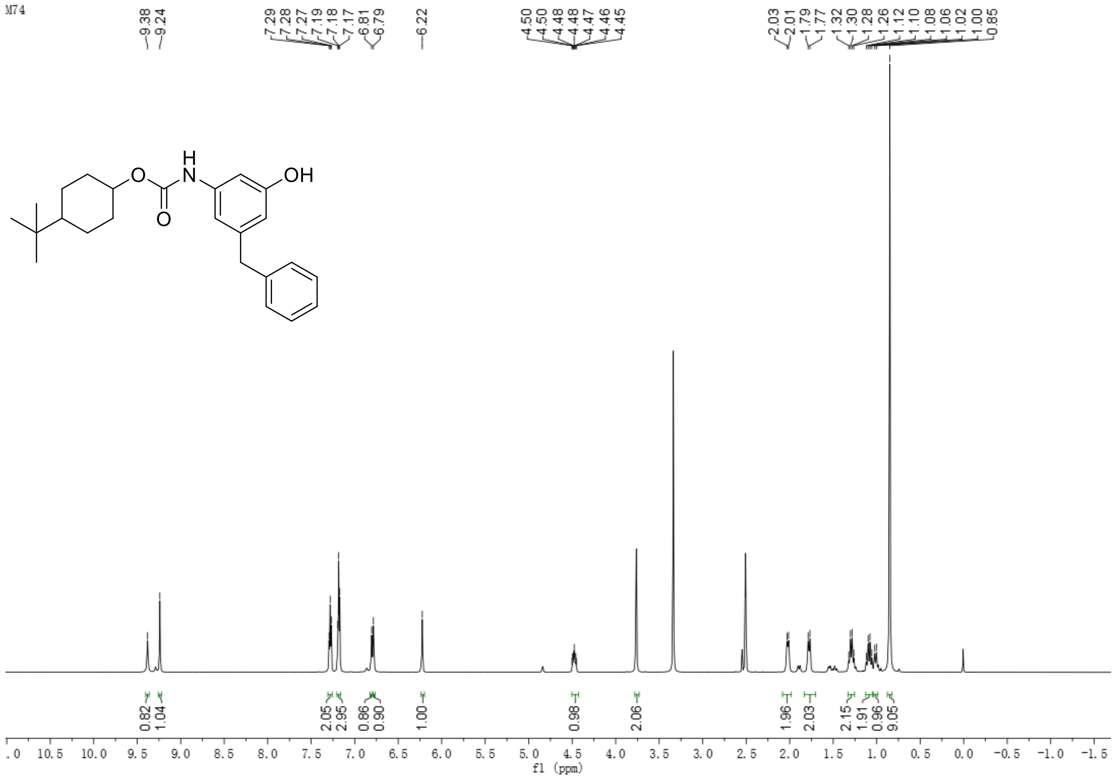
M74
9.38
9.24
7.29
7.28
7.27
7.19
7.18
7.17
6.81
6.79
6.22
4.50
4.50
4.48
4.48
4.47
4.46
4.45
2.03
2.01
1.79
1.77
1.32
1.30
1.28
1.26
1.12
1.10
1.08
1.06
1.02
1.00
0.85
H
N
O
O
O
OH
0.82
1.04
2.05
2.95
0.86
0.90
1.00
0.98
2.06
1.96
2.03
2.15
1.91
0.96
9.05
f1 (ppm)

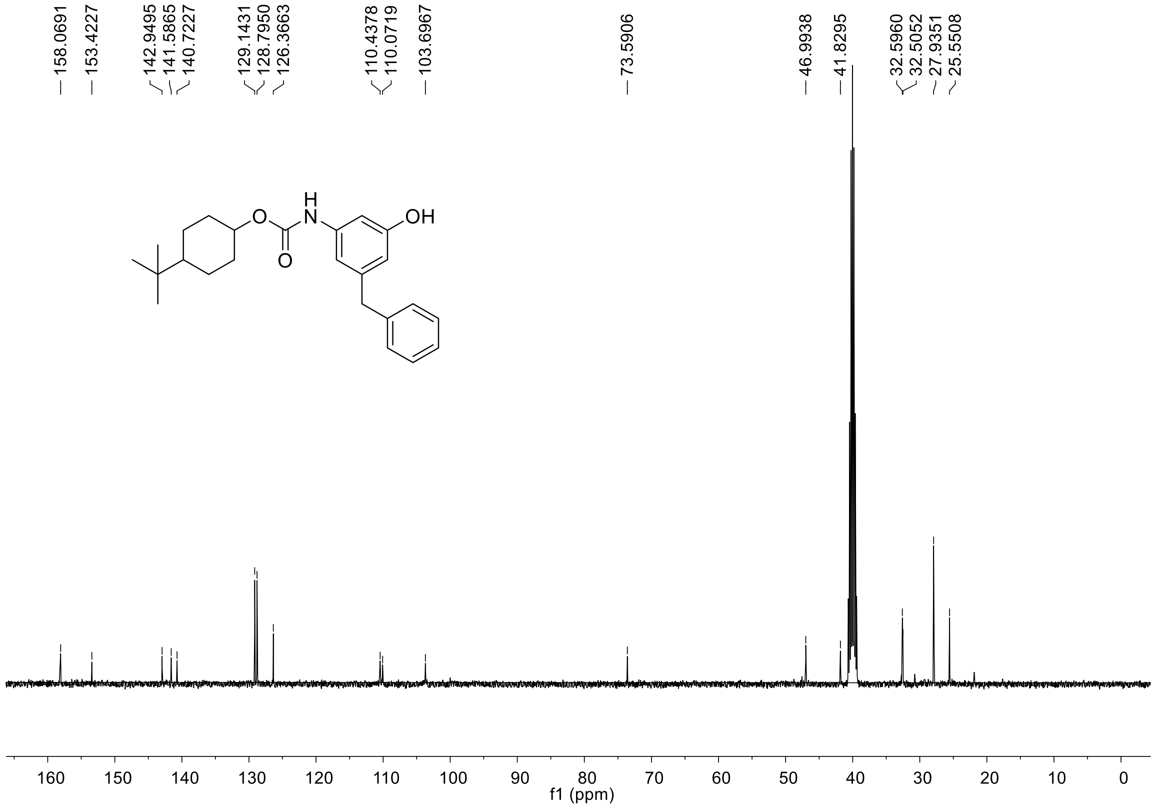
158.0691
153.4227
142.9495
141.5865
140.7227
129.1431
128.7950
126.3663
110.4378
110.0719
103.6967
73.5906
46.9938
41.8295
32.5960
32.5052
27.9351
25.5508
H
N
O
O
O
OH
f1 (ppm)

Compound **3n**

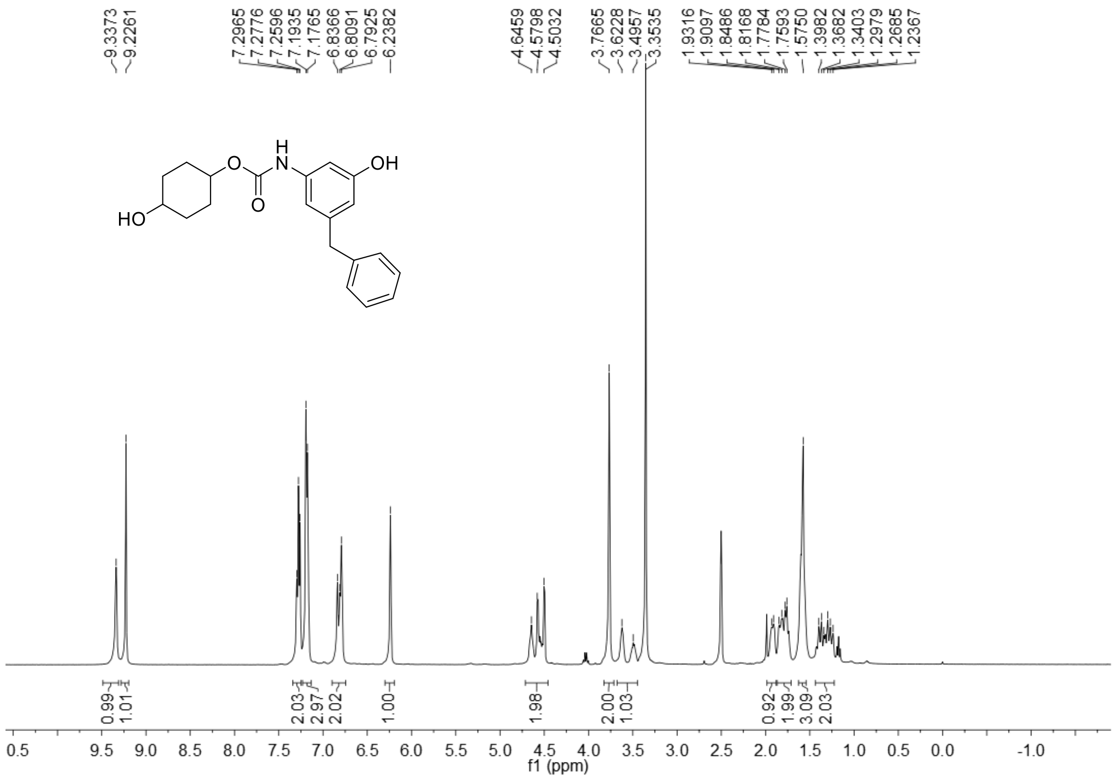

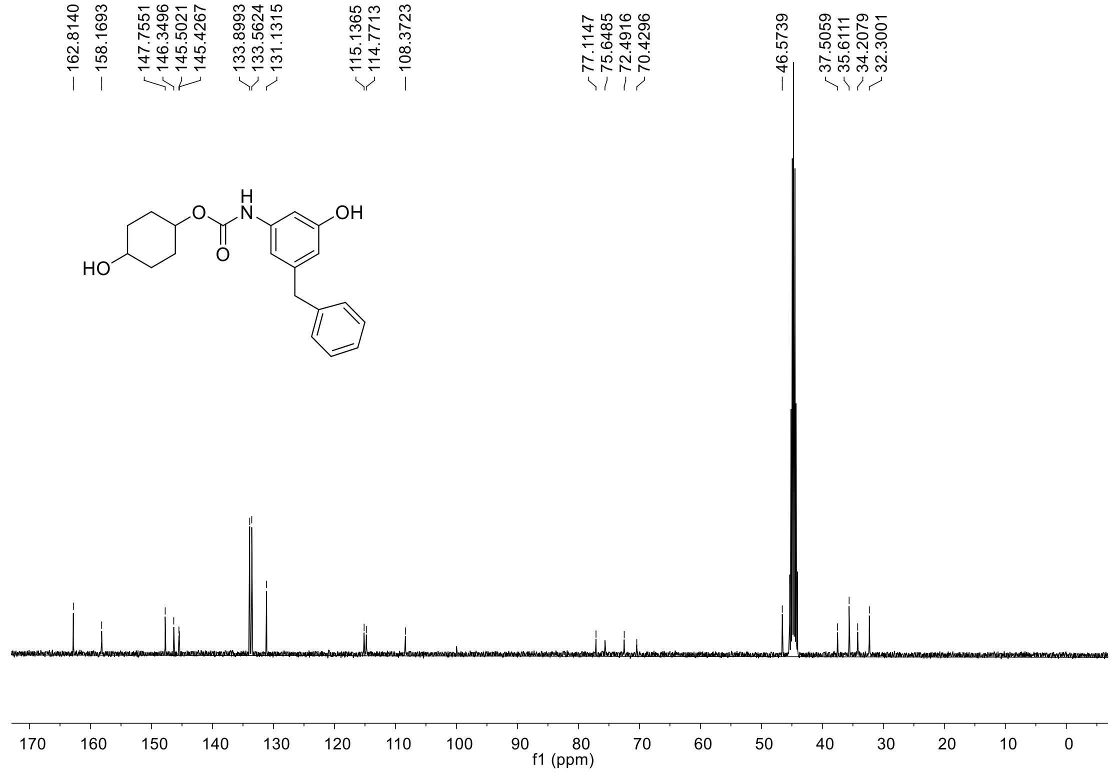

Compound **3o**

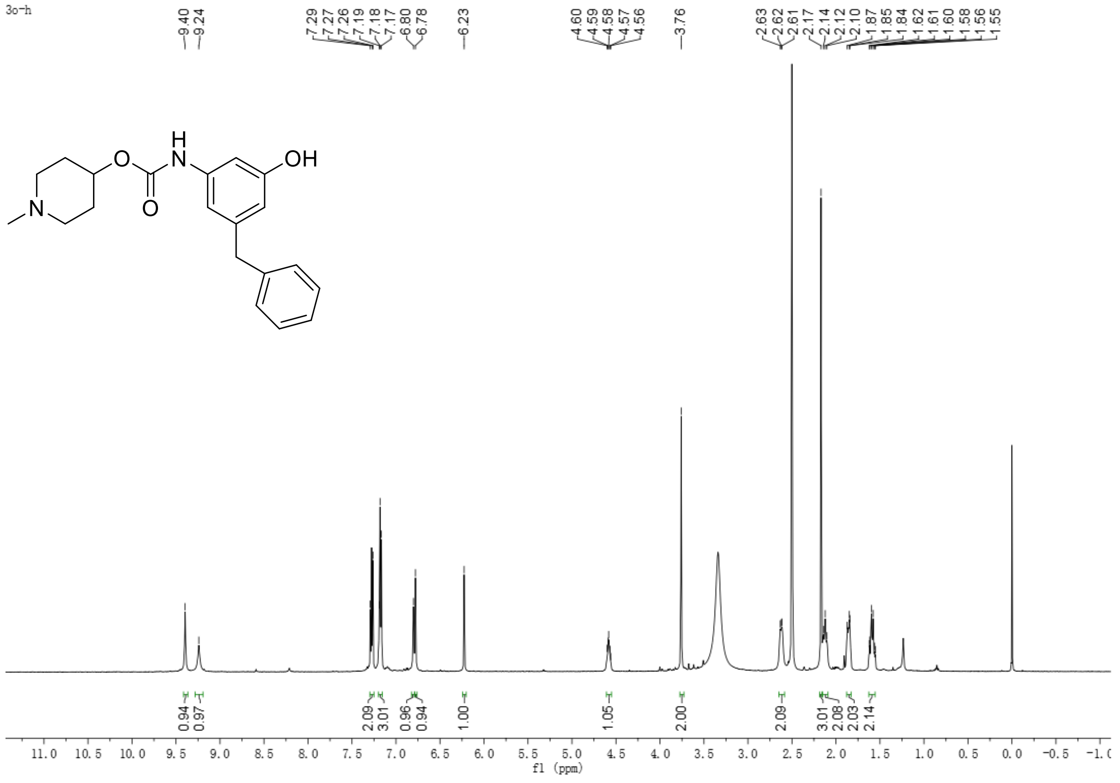
3o-h
9.40
9.24
7.29
7.27
7.26
7.19
7.18
7.17
6.80
6.78
6.23
4.60
4.59
4.58
4.57
4.56
3.76
2.63
2.62
2.61
2.17
2.14
2.12
2.10
1.87
1.85
1.84
1.62
1.61
1.60
1.58
1.56
1.55
OH
0.94
0.97
2.09
3.01
0.96
0.94
1.00
1.05
2.00
2.09
3.01
2.08
2.03
2.14
f1 (ppm)

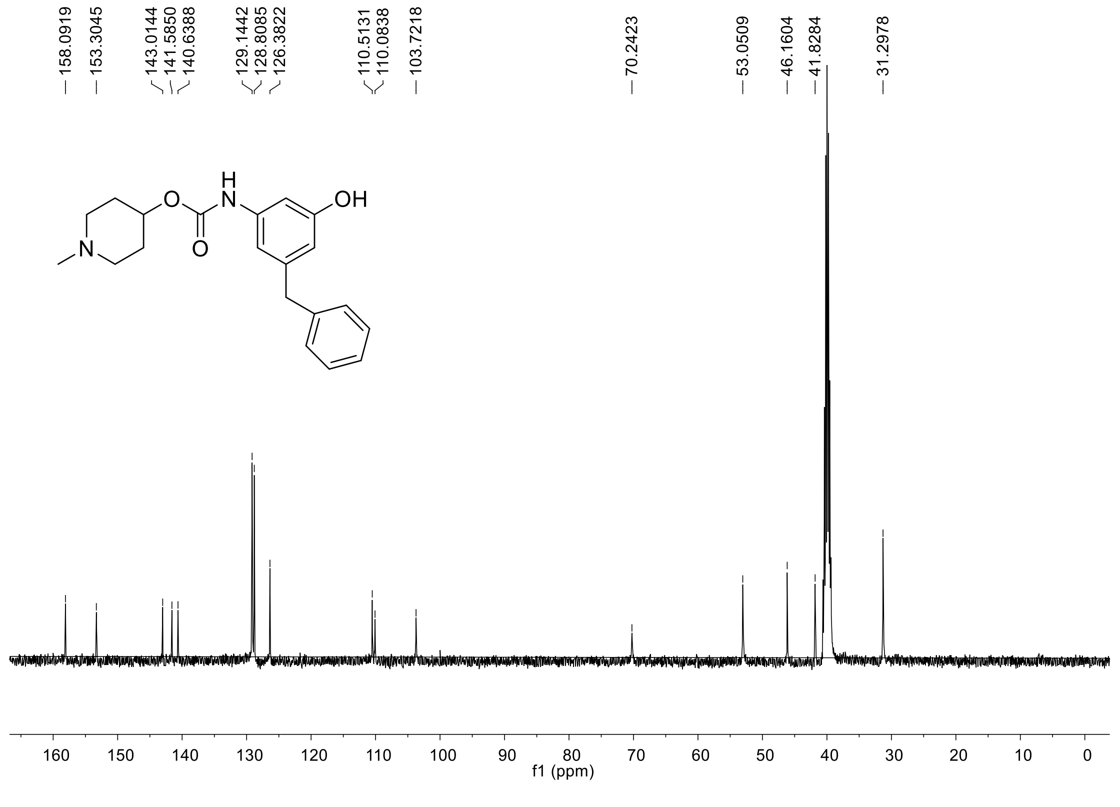
158.0919
153.3045
143.0144
141.5850
140.6388
129.1442
128.8085
126.3822
110.5131
110.0838
103.7218
70.2423
53.0509
46.1604
41.8284
31.2978
OH
f1 (ppm)

Compound **3p**

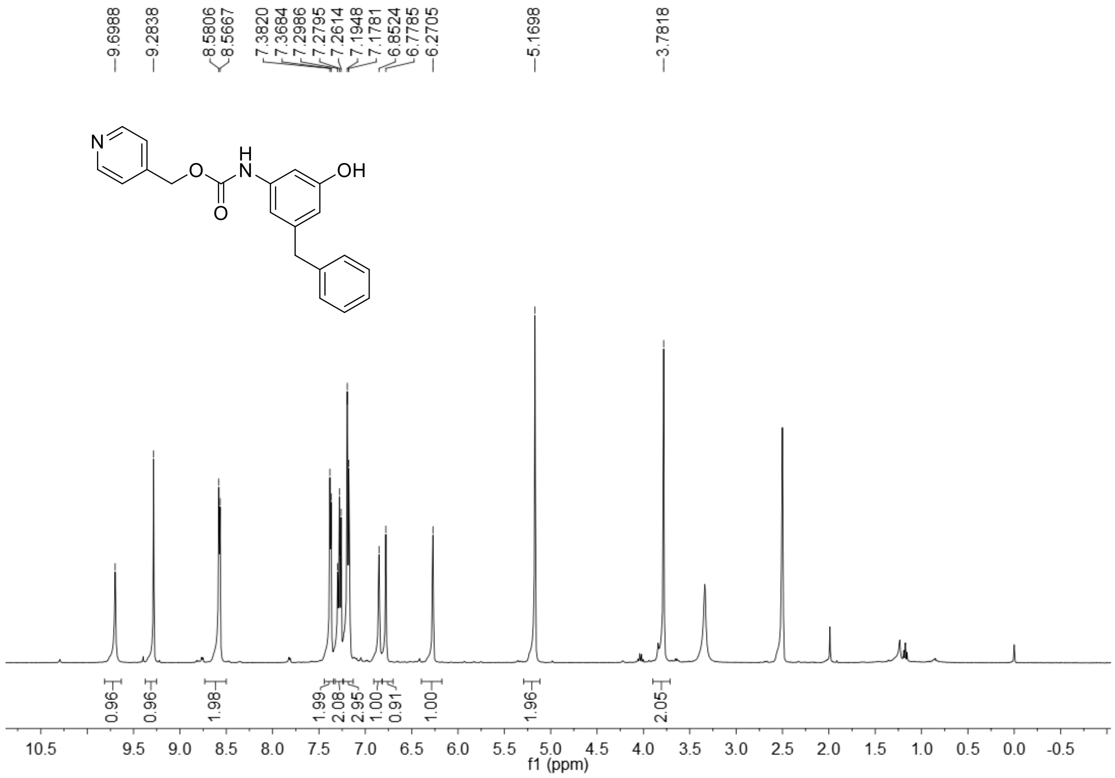
9.6988
9.2838
8.5806
8.5667
7.3820
7.3684
7.2986
7.2795
7.2614
7.1948
7.1781
6.8524
6.7785
6.2705
5.1698
3.7818
N
H
N
O
O
OH
0.96
0.96
1.98
1.99
2.08
2.95
1.00
0.91
1.00
1.96
2.05
10.5 9.5 9.0 8.5 8.0 7.5 7.0 6.5 6.0 5.5 5.0 4.5 4.0 3.5 3.0 2.5 2.0 1.5 1.0 0.5 0.0 -0.5
f1 (ppm)

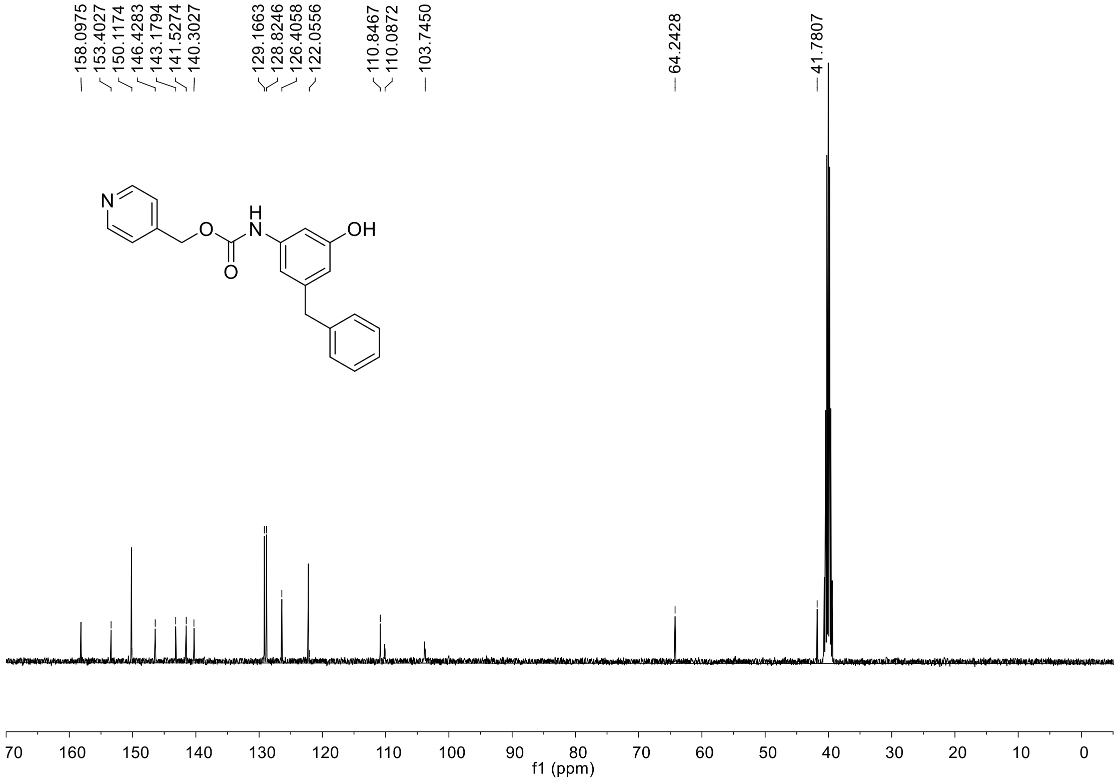
158.0975
153.4027
150.1174
146.4283
143.1794
141.5274
140.3027
129.1663
128.8246
126.4058
122.0556
110.8467
110.0872
103.7450
64.2428
41.7807
N
H
N
O
O
OH
70 160 150 140 130 120 110 100 90 80 70 60 50 40 30 20 10 0
f1 (ppm)

Compound **3q**

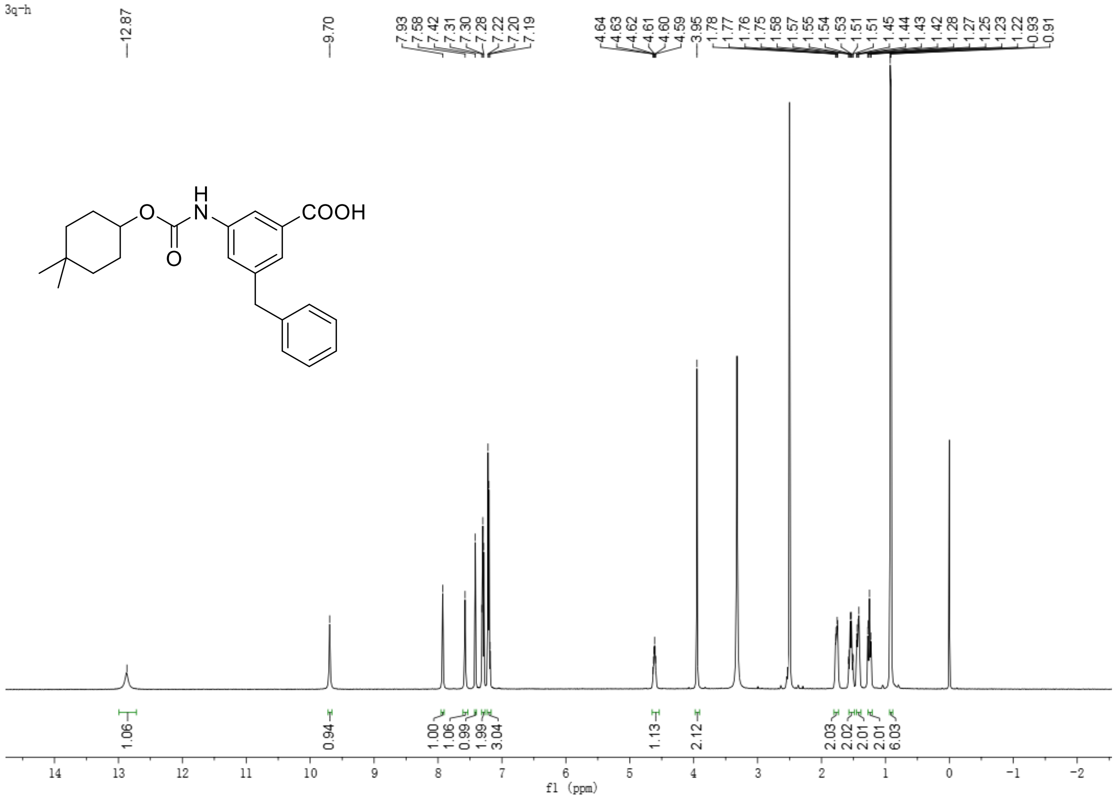

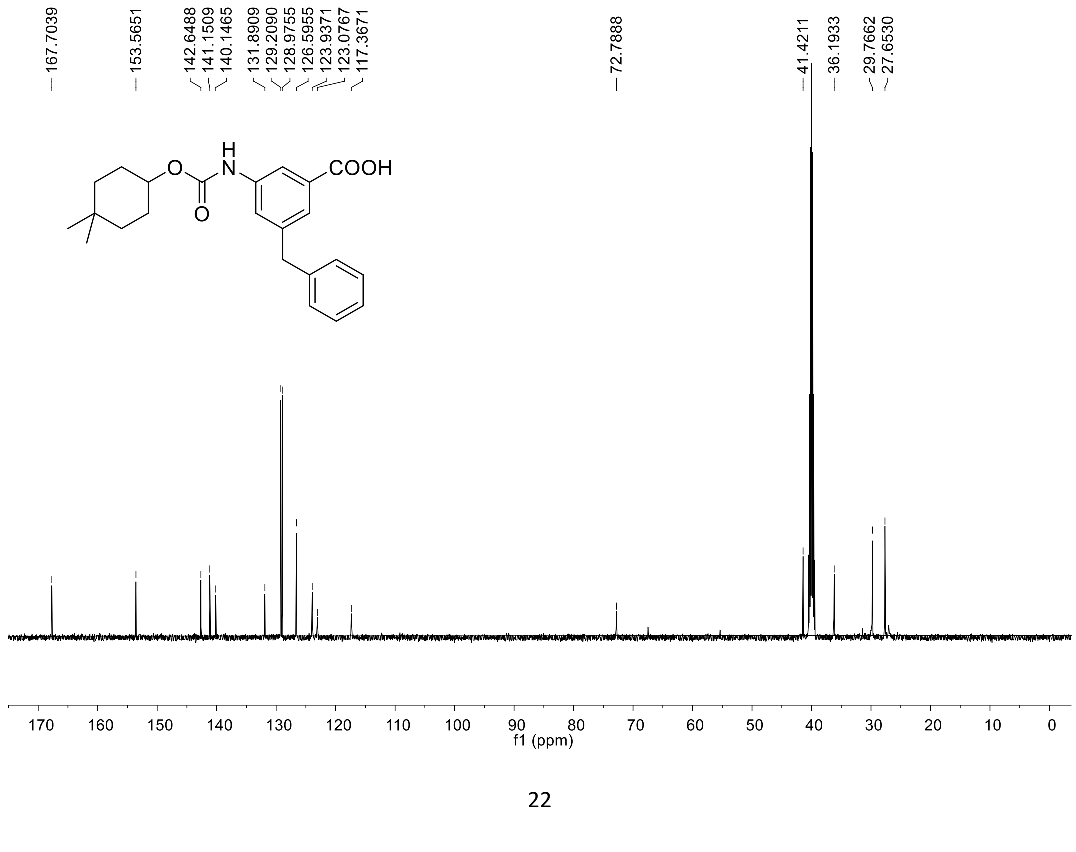

Compound **3r**

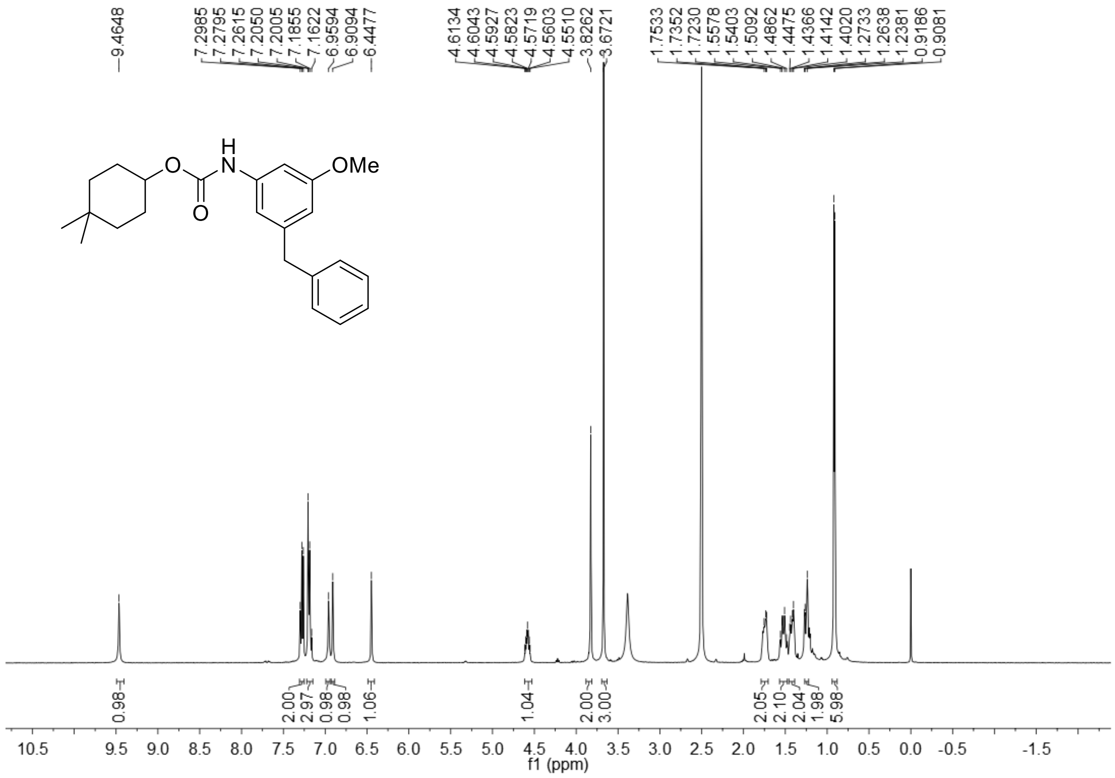

9.4648
7.2985
7.2795
7.2615
7.2050
7.2005
7.1855
7.1622
6.9594
6.9094
6.4477
4.6134
4.6043
4.5927
4.5823
4.5719
4.5603
4.5510
3.8262
3.6721
1.7533
1.7352
1.7230
1.5578
1.5403
1.5092
1.4862
1.4475
1.4366
1.4142
1.4020
1.2733
1.2638
1.2381
0.9186
0.9081
OMe
0.98
2.00
2.97
0.98
0.98
1.06
1.04
2.00
3.00
2.05
2.10
2.04
1.98
5.98
f1 (ppm)

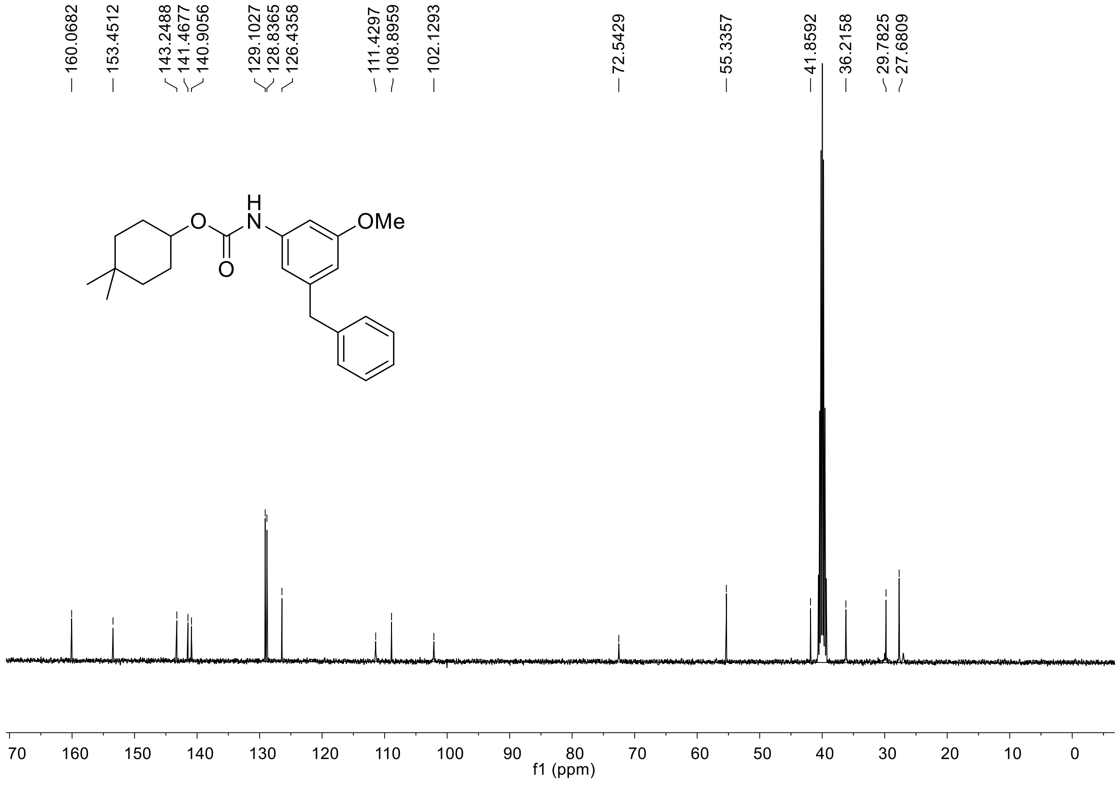

160.0682
153.4512
143.2488
141.4677
140.9056
129.1027
128.8365
126.4358
111.4297
108.8959
102.1293
72.5429
55.3357
41.8592
36.2158
29.7825
27.6809
OMe
f1 (ppm)
